# Global, regional, and national burden of atrial fibrillation and flutter associated with metabolic risk factors, 1990–2021

**DOI:** 10.3389/fcvm.2025.1578908

**Published:** 2025-09-08

**Authors:** Lu Wang, Xinghua Qin, Linyan Jin, Haoyu Gong, Chong Gao, Hongtao Wang, Peng Liu

**Affiliations:** ^1^ Department of Endocrinology, The First Affiliate Hospital of Xi’an Jiaotong University, Xi’an, China; ^2^ Xi’an Key Laboratory of Special Medicine and Health Engineering, School of Life Sciences, Northwestern Polytechnical University, Xi’an, Shaanxi, China; ^3^ Department of Cardiology, The Second Affiliate Hospital of Xi’an Jiaotong University, Xi’an, China

**Keywords:** atrial fibrillation/flutter, metabolic risk factors, global burden of disease, disability-adjusted life years, estimated annual percentage changes

## Abstract

**Objectives:**

Atrial fibrillation/flutter (AF/AFL) is increasingly correlated with metabolic risk factors. This study analyzed mortality, disability-adjusted life years (DALYs), annual percentage changes (APCs), and estimated annual percentage changes (EAPCs), stratified by age, sex, Socio-demographic Index (SDI), and region, to evaluate the global burden of AF/AFL linked to metabolic risk factors.

**Methods:**

Data on prevalence, incidence, mortality, DALYs, and age-standardized rates (ASRs) for AF/AFL associated with metabolic risk factors were extracted from the Global Burden of Disease (GBD) 2021 study. EAPCs were used to assess temporal trends in ASRs.

**Results:**

In 2021, metabolic risk factors were correlated with approximately 117,012 deaths and 2,881,800 DALYs globally due to AF/AFL, reflecting increases of 196.08% and 154.51%, respectively, compared to 1990. The burden varied considerably by sex, SDI, and region. Women, older adults, and high-SDI regions exhibited higher burdens, while men, younger individuals, and low-SDI regions showed more rapid increases.

**Conclusions:**

Metabolic risk factors are strongly correlated with the global burden of AF/AFL. Strengthening cost-effective interventions targeting these modifiable risks is essential—particularly in regions facing high or rapidly growing burdens.

## 1 Introduction

Atrial fibrillation/flutter (AF/AFL) is one of the most common arrhythmias encountered in clinical practice, with its incidence progressively rising due to an aging population and the increasing complexity of comorbid conditions (1, 2). AF/AFL not only causes symptoms such as palpitations and chest tightness, leading to a reduced quality of life for patients, but also significantly increases the risk of stroke, heart failure, and mortality (3, 4). The intricate pathological mechanisms of AF/AFL, coupled with numerous risk factors, pose challenges in its prevention and treatment, establishing it as a global health imperative (5).

AF/AFL is strongly associated with metabolic dysfunction (6, 7). A key piece of evidence is that the rapid contraction of the atrial myocardium during AF/AFL leads to marked fluctuations in energy supply and demand (6). Moreover, numerous studies have consistently shown that metabolic factors—including obesity, insulin resistance, and diabetes mellitus—significantly increase the risk of developing AF/AFL (7). Notably, in contrast to non-modifiable risk factors such as age and sex, numerous studies have indicated that addressing modifiable metabolic risk factors can substantially reduce both the incidence of AF/AFL and its related complications (5). However, comprehensive epidemiological data quantifying the burden of AF/AFL specifically in relation to metabolic risk factors remain limited.

An accurate assessment of the global burden of AF/AFL related to metabolic risk factors is essential for developing effective prevention and control strategies. The GBD database offers a comprehensive understanding of the global distribution, temporal trends, and influencing factors of AF/AFL, thereby providing valuable guidance for clinical management and policy-making (8). In this study, we used data from GBD 2021 to evaluate the global burden of AF/AFL correlated with metabolic risk factors. We also analyzed temporal trends from 1990 to 2021, stratified by geographic region, sex, age group, and development status. Our findings complement existing evidence and provide important insights to inform prevention and treatment strategies for AF/AFL.

## 2 Methods

### 2.1 Data sources

The raw data were obtained from the GBD 2021 database (https://vizhub.healthdata.org/gbd-results/). The detailed methodology and analytical framework used in GBD 2021 have been published in prior reports. The GBD 2021 dataset categorizes AF/AFL-related risk factors into three main groups: metabolic, behavioral, and environmental/occupational. Detailed definitions of metabolic risk factors are provided in the Supplementary Material (Online 1, Supplementary Table 1S).

### 2.2 Study design

Annual DALYs and corresponding ASRs related to the burden of AF/AFL associated with metabolic risk factors were extracted and analyzed. Mortality was defined as the number of deaths per 100,000 individuals. ASRs were calculated as weighted averages of age-specific rates, adjusted for population size and age distribution. To estimate the geographic distribution of disease burden, data from the GBD database were used, encompassing 204 countries and territories, 5 SDI quintiles, and 21 GBD-defined regions.

### 2.3 Definitions

The International Classification of Diseases and Injuries (ICD-9 and ICD-10) was used to identify AF/AFL. Cardiovascular conditions coded as 427.3–427.32 in ICD-9 and I48–I48.92 in ICD-10 were classified as AF/AFL in this study. AF/AFL cases included in the GBD 2021 study were derived from a wide range of data sources, including published literature, hospital and outpatient records, insurance claims databases, and epidemiological surveys. These sources employed varying diagnostic approaches based on local clinical practices and resource availability. Although specific diagnostic modalities-such as 12-lead electrocardiograms (ECG), Holter monitoring, or other ambulatory techniques-were not uniformly applied across all data sources, most high-quality datasets relied on ECG confirmation for diagnosis.

The SDI is a composite measure reflecting the socioeconomic conditions that influence health outcomes across countries and regions, ranging from 0 (lowest) to 1 (highest). Countries and regions were grouped into five SDI categories—Low, Low-middle, Middle, High-middle, and High—to examine the association between AF/AFL burden and socioeconomic development.

### 2.4 Statistical analysis

The GBD 2021 analysis employed a comparative risk assessment framework structured around a hierarchy of modifiable and often interrelated risks. For each risk–outcome pair, relative risks (RRs), summary exposure values (SEVs), and theoretical minimum risk exposure levels (TMRELs) were calculated. To address the complex interplay among overlapping risk factors, the GBD framework employed mediation adjustments to distinguish between direct and indirect effects. Additionally, the newly developed Burden of Proof Risk Function (BPRF) provided conservative estimates of risk–outcome relationships by evaluating evidence consistency and heterogeneity. All estimates represent the mean of 500 simulation draws, with 95% uncertainty intervals defined by the 2.5th and 97.5th percentiles (9).

The temporal trends in the AF/AFL burden related to metabolic risk factors were calculated using the EAPC along with its 95% confidence interval (95% CI) (10). The EAPC values and their 95% CIs for each identified trend were estimated by fitting a regression line to the natural logarithm of the rates, using calendar year as the independent variable. An ASR was considered to be increasing if both the EAPC and its lower 95% CI were greater than zero. Conversely, it was considered to be decreasing if both the EAPC and its upper 95% CI were below zero. A smoothing splines model was applied to evaluate the relationship between EAPCs and SDI. All statistical analyses and visualizations were performed using R software (Version 4.1.2) and JD_GBDR. A *p*-value of less than 0.05 was regarded as statistically significant.

## 3 Results

### 3.1 Global AF/AFL burden associated with metabolic risk factors in 2021

**Figure 1:**
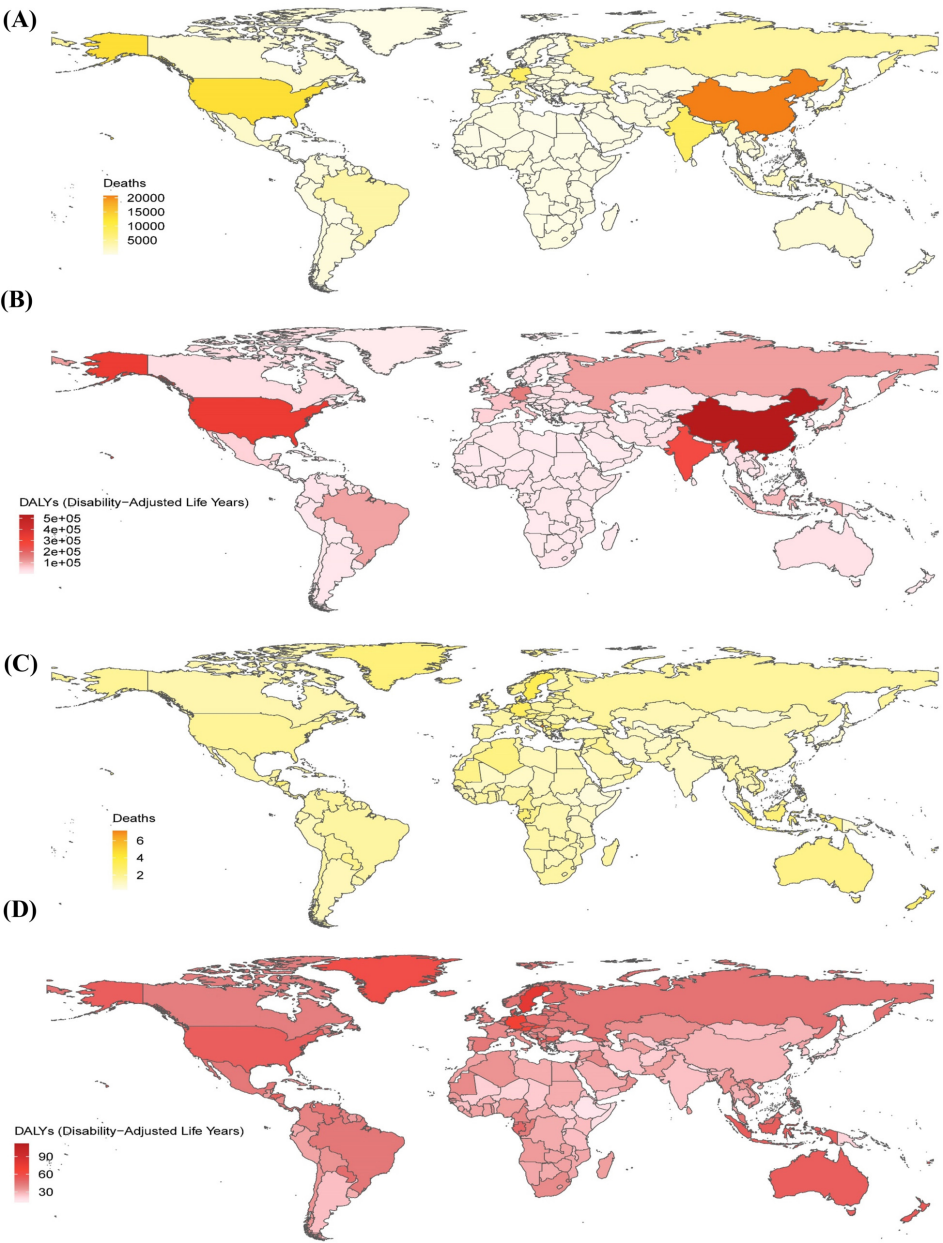
The global metabolic risk-related burden of atrial fibrillation/flutter across 204 countries and territories in 2021. The number of deaths **(A)** and disability-adjusted life years **(B)** in 204 countries and territories in 2021. The age-standardized *r*ates (per 100,000 persons) of deaths **(C)** and disability-adjusted life years **(D)** in 204 countries and territories in 2021. DALYs, disability-adjusted life years.

In 2021, AF/AFL related to metabolic risk factors was responsible for an estimated 117,012 deaths (95% UI: 53,219.12–180,984.63) and 2,881,800.45 DALYs globally (95% UI: 1,296,238.10–4,507,684.79), representing increases of 196.08% and 154.51%, respectively, compared to 1990 (Tables 1, 2). Regions with the highest mortality included East Asia, high-income North America, South Asia, and Western Europe, with Western Europe reporting the highest number of deaths (25,859.45; 95% UI: 11,813.39–39,983.81). These regions also showed a high DALY burden, with East Asia reporting the highest value (551,927.47; 95% UI: 223,379.50–907,787.91). Among affected countries, China, the United States, India, and Germany had the highest overall burden. Specifically, China recorded 531,011.93 DALYs (95% UI: 214,701.35–876,899.81) and 20,752.99 deaths (95% UI: 8,276.98–34,233.63) associated with metabolic risk factors (Figures 1A,B; Supplementary Tables S2, S3).

**Table 1:** Numbers and age standardized deaths rates of atrial fibrillation/flutter related to metabolic risk factors in 1990 and 2021, and their percentage change of death numbers, estimated annual percentage changes in age standardized death rate from 1990 to 2021, by sex and socio-demographic index region.

Category	1990		2021			
	Number of DALYs (95% UI)	ASR of DALYs per 100,000 people (95% UI)	Number of DALYs (95% UI)	ASR of DALYs per 100,000 people (95% UI)	PC number of DALYs 1990–2021 (95% UI)	EAPC of ASR of DALYs 1990–2021 (95% CI)
Global	1,132,279.61 (468,240.81–1,783,642.78)	34.22 (14.16–53.90)	2,881,800.45 (1,296,238.10–4,507,684.79)	34.94 (15.64–54.66)	154.51 (135.27–186.45)	0.00(−0.03 to –0.03)
Sex						
Male	505,413.26 (198,639.35–818,479.21)	35.51 (14.05–56.86)	1,332,970.46 (586,629.25–2,118,474.31)	36.91 (16.03–58.83)	164.26 (144.55–205.53)	0.13 (0.12–0.15)
Female	627,866.35 (269,503.64–976,825.53)	32.78 (14.05–50.65)	1,548,829.99 (715,458.95–2,393,476.30)	33.00 (15.23–50.93)	146.68 (125.67–177.04)	−0.09(−0.14 to −0.05)
SDI regions						
Low SDI	33,803.96 (11,662.21–58,855.17)	20.60 (7.18–35.76)	96,101.45 (34,540.95–162,777.16)	25.15 (9.19–42.64)	184.29 (148.61–225.96)	0.76 (0.68–0.84)
Low-middle SDI	106,829.06 (39,688.10–178,162.25)	23.23 (8.65–38.93)	365,398.13 (148,611.51–578,845.63)	30.94 (12.57–48.84)	242.04 (203.63–298.40)	0.99 (0.95–1.02)
Middle SDI	198,415.55 (75,104.03–330,762.09)	26.39 (9.94–43.34)	762,509.43 (320,628.02–1,216,525.37)	32.45 (13.50–52.12)	284.30 (236.60–355.18)	0.67 (0.62–0.72)
High-middle SDI	291,594.64 (122,343.02–456,561.77)	34.66 (14.55–53.56)	671,753.90 (307,337.35–1,049,469.50)	34.51 (15.78–53.84)	130.37 (110.23–163.51)	−0.05(−0.10 to −0.00)
High SDI	499,919.49 (214,996.38–776,371.94)	44.86 (19.32–69.59)	982,833.44 (468,581.50–1,530,934.85)	41.67 (19.79–64.99)	96.60(77.35–133.53)	−0.40(−0.48 to −0.31)

**DALYs, disability−adjusted life years; EAPC,:** estimated annual percentage changes; SDI, socio−demographic index; ASR, age standardized rate; UI, uncertainty interval; PC, percentage change; CI, confidence interval.

**Table 2:** Numbers and age standardized disability-adjusted life years rates of atrial fibrillation/flutter related to metabolic risk factors in 1990 and 2021, and their percentage change of disability-adjusted life years numbers, estimated annual percentage changes in age standardized rate from 1990 to 2021, by sex and socio-demographic index region.

Category	1990		2021			
	Number of DALYs (95% UI)	ASR of DALYs per 100,000 people (95% UI)	Number of DALYs (95% UI)	ASR of DALYs per 100,000 people (95% UI)	PC number of DALYs 1990–2021 (95% UI)	EAPC of ASR of DALYs 1990–2021 (95% CI)
Global	1,132,279.61 (468,240.81–1,783,642.78)	34.22 (14.16–53.90)	2,881,800.45 (1,296,238.10–4,507,684.79)	34.94 (15.64–54.66)	154.51 (135.27–186.45)	0.00 (−0.03 to –0.03)
Sex						
Male	505,413.26 (198,639.35–818,479.21)	35.51 (14.05–56.86)	1,332,970.46 (586,629.25–2,118,474.31)	36.91 (16.03–58.83)	164.26 (144.55–205.53)	0.13 (0.12–0.15)
Female	627,866.35 (269,503.64–976,825.53)	32.78 (14.05–50.65)	1,548,829.99 (715,458.95–2,393,476.30)	33.00 (15.23–50.93)	146.68 (125.67–177.04)	−0.09(−0.14 to −0.05)
SDI regions						
Low SDI	33,803.96 (11,662.21–58,855.17)	20.60 (7.18–35.76)	96,101.45 (34,540.95–162,777.16)	25.15 (9.19–42.64)	184.29 (148.61–225.96)	0.76 (0.68–0.84)
Low-middle SDI	106,829.06 (39,688.10–178,162.25)	23.23 (8.65–38.93)	365,398.13 (148,611.51–578,845.63)	30.94 (12.57–48.84)	242.04 (203.63–298.40)	0.99 (0.95–1.02)
Middle SDI	198,415.55 (75,104.03–330,762.09)	26.39 (9.94–43.34)	762,509.43 (320,628.02–1,216,525.37)	32.45 (13.50–52.12)	284.30 (236.60–355.18)	0.67 (0.62–0.72)
High-middle SDI	291,594.64 (122,343.02–456,561.77)	34.66 (14.55–53.56)	671,753.90 (307,337.35–1,049,469.50)	34.51 (15.78–53.84)	130.37 (110.23–163.51)	−0.05(−0.10 to −0.00)
High SDI	499,919.49 (214,996.38–776,371.94)	44.86 (19.32–69.59)	982,833.44 (468,581.50–1,530,934.85)	41.67 (19.79–64.99)	96.60 (77.35–133.53)	−0.40(−0.48 to −0.31)

**DALYs, disability−adjusted life years; EAPC,:** estimated annual percentage changes; SDI, socio−demographic index; ASR, age standardized rate; UI, uncertainty interval; PC, percentage change; CI, confidence interval.

In 2021, in terms of age-standardized death rates (ASDRs), Australasia, Western Europe, high-income North America, and Central Europe exhibited the highest rates. Australasia reported the highest ASDR (2.28; 95% UI: 1.10–3.50). These regions also had elevated DALY rates, with Australasia again reporting the highest value (52.62; 95% UI: 24.91–81.13). Among all countries, Montenegro ranked highest in both ASDR (7.15; 95% UI: 3.54–11.12) and DALY rate (112.49; 95% UI: 56.04–173.21), as shown in Figures 1C,D.

### 3.2 Temporal trends in the burden of AF/AFL associated with metabolic risk factors

From 1990 to 2021, the burden of AF/AFL exhibited diverse absolute changes across regions. Overall, most regions experienced a substantial increase in deaths, with the highest increases observed in South Asia and the Maldives, at 416.66% and 637.60%, respectively. However, some countries demonstrated stable mortality trends or exhibited only marginal increases (less than 50%), such as Finland, Grenada, and Norway. Notably, Niue reported a 2.94% decrease in deaths (Supplementary Figure S1A). DALY counts related to metabolic risk factors followed similar trends, with the highest increases recorded in Andean Latin America (371.07%) and Qatar (690.60%) (Supplementary Figure S1B).

Globally, the ASDR for AF/AFL related to metabolic risk factors increased from 1990 to 2021, with an EAPC of 0.07 (95% CI: 0.03–0.11). In contrast, age-standardized DALY rates showed no significant change, with an EAPC of 0.00 (95% CI: −0.03 to 0.03). South Asia exhibited the most pronounced increase in ASDR, with an EAPC of 1.85 (95% CI: 1.64–2.06), whereas high-income Asia Pacific experienced the largest decline (EAPC: −2.05; 95% CI: −2.47 to −1.62). Among GBD regions, Andean Latin America recorded the highest increase in DALY ASRs (EAPC: 1.61; 95% CI: 1.46–1.76), while high-income Asia Pacific had the greatest decrease (EAPC: −1.66; 95% CI: −1.98 to −1.35). At the national level, the United Arab Emirates showed the most substantial increase in ASDR (EAPC: 3.27; 95% CI: 2.24–4.30), whereas Finland reported the largest decline (EAPC: −2.27; 95% CI: −2.52 to −2.02). Oman had the highest EAPC for DALY ASRs (2.80; 95% CI: 2.52–3.08), while Singapore exhibited the greatest reduction (EAPC: −2.29; 95% CI: −2.58 to −2.01), as shown in Supplementary Figures S1C, S1D.

### 3.3 Disparities across SDI quintiles

The burden of AF/AFL linked to metabolic risk factors varied across the 5 SDI quintiles. From 1990 to 2021, all SDI quintiles showed increases in both deaths and DALYs (Supplementary Figure S2). In 2021, the high SDI quintile bore the greatest burden, accounting for 44,391 deaths and 982,833 DALYs. In contrast, the lowest burden was observed in the low SDI quintile, with 3,181 deaths and 96,101 DALYs. Between 1990 and 2021, the middle SDI quintile experienced the most rapid increases in deaths and DALYs, with respective growth rates of 348.76% and 284.30% (Tables 1, 2). The highest ASRs for deaths (1.65 per 100,000) and DALYs (41.67 per 100,000) were recorded in the high SDI quintile. While ASRs for deaths and DALYs in the high SDI quintile declined over time, those in other quintiles continued to increase (Supplementary Figure S3).

The association between the AF/AFL burden linked to metabolic risk factors and the SDI was also examined. Overall, the relationship between SDI and both deaths and DALYs followed an asymmetrical, inverted V-shaped pattern (Supplementary Figure S4). Additionally, ASRs for deaths and DALYs showed no significant association with the EAPC. However, an inverse association was observed between EAPC and SDI, as shown in Supplementary Figure S5.

### 3.4 Heterogeneities in AF/AFL burden by sex and age associated with metabolic risk factors

The burden of AF/AFL linked to metabolic risk factors demonstrated significant sex-related disparities, with women experiencing substantially higher numbers of deaths and DALYs globally compared to men. The ASDR was higher among females, whereas males exhibited a higher DALYs. Among females, the age-standardized DALY rate declined, with an EAPC of −0.09 (95% CI: −0.14 to −0.05). In contrast, ASRs for both mortality and DALYs showed a modest increase among males (mortality: 0.22; 95% CI: 0.20–0.25; DALYs: 0.13; 95% CI: 0.12–0.15), as presented in Tables 1, 2. In both 1990 and 2021, Western Europe recorded the highest number of deaths related to AF/AFL linked to metabolic risk factors across both sexes. Additionally, Western Europe reported the highest DALY counts for both males and females. However, in 2021, East Asia surpassed Western Europe in female DALY numbers, as illustrated in Supplementary Figure S6.

**Figure 2:**
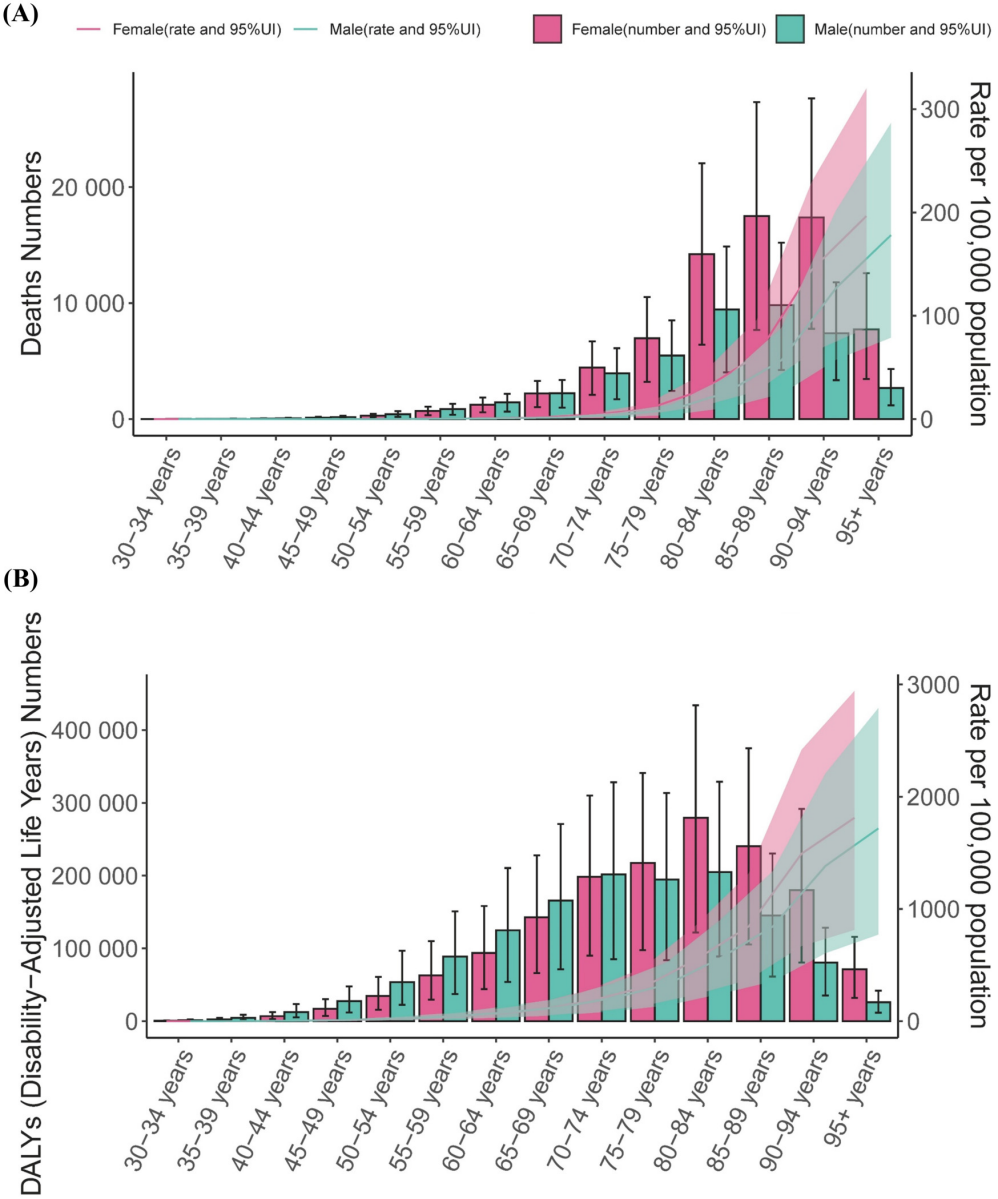
Age-specific numbers and rates of deaths and disability-adjusted life years associated with atrial fibrillation and flutter by sex in 2021. Age-specific numbers **(A)** and rates **(B)** of deaths and DALYs due to AF/AFL in 2021, stratified by sex. Error bars represent the 95% UIs for absolute numbers. Shaded areas indicate the 95% UIs for age-specific rates. DALYs, disability-adjusted life years; UI, uncertainty interval.

From 1990 to 2021, deaths and DALYs of AF/AFL associated with metabolic risk factors increased across all age groups and sexes. This trend was particularly pronounced among older adults, especially those aged over 80 years (Supplementary Figure S7). Furthermore, the rate of death and DALY remained relatively stable across most age groups, except for a marked increase among individuals aged ≥80 years, affecting both sexes (Supplementary Figure S8). In 2021, both the number and rate of deaths and DALYs rose progressively with age in males and females (Figure 2).

### 3.5 Proportion and temporal patterns of metabolic risk factors associated with AF/AFL burden

**Figure 3:**
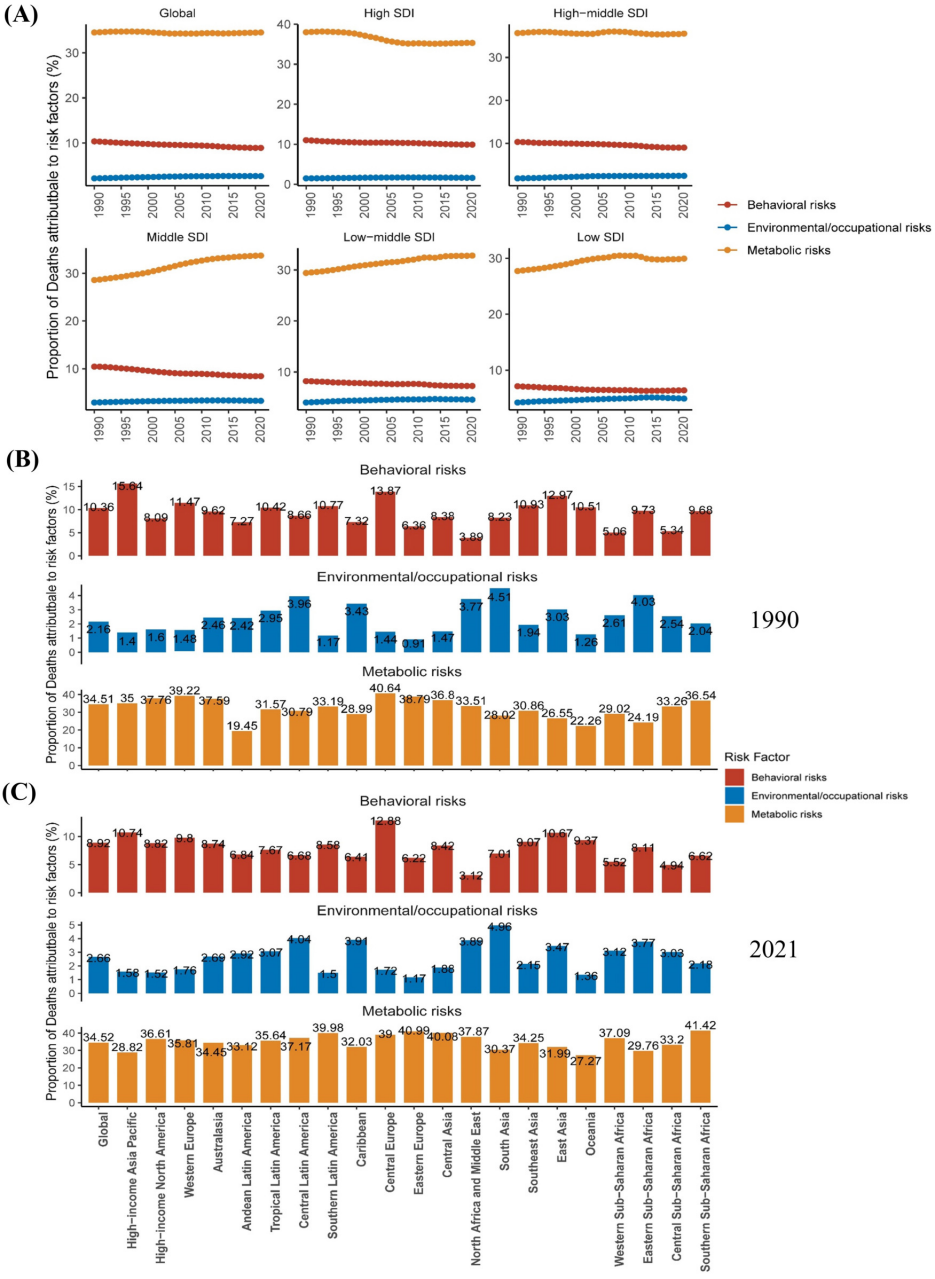
Risk factors and temporal trends associated with atrial fibrillation/flutter. **(A)** Trends in the proportion of AF/AFL deaths correlated with various risk factors from 1990 to 2021. **(B,C)** Percentage contributions of major risk factors to age-standardized deaths associated with AF/AFL across 21 regions in 1990 and 2021. AF/AFL, atrial fibrillation/flutter.

Between 1990 and 2021, metabolic risk factors were most strongly correlated with AF/AFL-related deaths and DALYs, followed by behavioral and environmental or occupational risks. The findings suggest that the correlation between metabolic risk factors and AF/AFL-related mortality remained relatively stable at the global level during this period, with a declining trend observed in high-SDI regions. In contrast, an increasing trend was observed in regions with low, low-middle, and middle SDI levels (Figure 3A). In 1990, Central Europe had the highest contribution of metabolic risk factors to AF/AFL-related mortality (40.64%), whereas by 2021, Southern Sub-Saharan Africa had become the leading contributor (41.42%) (Figures 3B,C). The impact of metabolic risk factors on AF/AFL-related mortality also varied by age, increasing progressively with advancing age and peaking among individuals aged 70–74 years (Supplementary Figure S9).

## 4 Discussion

The association between AF/AFL and metabolic factors is well-established. This study offers a comprehensive assessment of the global burden of AF/AFL associated with metabolic risk from 1990 to 2021, considering sex, age, geographic region, and socioeconomic development status. While previous studies using GBD database resources have examined similar aspects, a thorough and updated analysis remains valuable. Our results indicate that metabolic risk factors are strongly correlated with AF/AFL-related mortality and DALYs over the past three decades, and this correlation appears to be influenced by multiple demographic and regional factors. These findings suggest that improvements in metabolic health may have meaningful implications for AF/AFL prevention efforts.

The complex mechanisms and multifactorial etiology of AF present substantial challenges for effective management (11). Metabolic disorders are recognized as significant risk factors for AF (5). Atrial metabolic remodeling plays a key role in the pathophysiology of AF and has been identified as a potential target for therapeutic intervention (5, 6). In the GBD study, metabolic risk factors encompass high fasting plasma glucose, elevated low-density lipoprotein cholesterol, high systolic blood pressure, increased body mass index, low bone mineral density, and kidney dysfunction (9, 12). Growing evidence indicates that active management of metabolic risk factors may help reduce the risk of AF/AFL onset and progression. Accordingly, addressing metabolic risk factors has become an important component of AF/AFL treatment strategies (5). Nonetheless, the relationship between AF/AFL and metabolic risk factors is not yet fully understood, underscoring the need for further research to explore mechanisms beyond those addressed in current clinical guidelines.

The incidence of AF and its associated complications is significantly influenced by sex and age (13, 14). Previous studies have shown that women have a relatively lower prevalence of AF/AFL compared to men. Although this study also found that age-standardized rates of death and DALYs have increased among men and decreased among women, the overall disease burden—reflected by total DALYs and mortality—remains higher in women (15). Similarly, deaths and DALYs linked to metabolic risk factors in AF/AFL were more frequently observed in women than in men. Evidence suggests that women are more likely to present with atypical symptoms, exhibit a higher risk of stroke, and report a lower quality of life due to AF/AFL. These disparities may be attributed to a combination of biological and hormonal factors, as well as potential systemic biases within research and clinical settings (16). The precise molecular mechanisms underlying these sex-based differences remain unclear and warrant further investigation. Clinical management strategies for AF/AFL—particularly in women—should emphasize rhythm control and complication prevention.

The influence of age on AF risk is well documented, with the highest burden observed among individuals aged over 80 years—a rapidly expanding demographic group (15). Consistent with previous findings, our study demonstrates a strong correlation between advancing age and increased AF/AFL-related mortality and DALYs associated with metabolic risk factors in both sexes. The number and rate of deaths and DALYs linked to metabolic risk factors were highest in individuals over 80 years, showing a markedly rising trend from 1990 to 2021. Aging not only increases vulnerability to AF but is also associated with the development of other cardiovascular diseases. Furthermore, the proportion of ischemic stroke and systemic embolism related to AF rises with age (17, 18). In contrast, the incidence of bleeding complications during anticoagulant therapy is more frequent among older adults. Despite this, elderly patients are often underrepresented in clinical trials, and the most appropriate treatment strategies remain uncertain (18, 19). As a result, designing effective therapeutic approaches for older adults with AF/AFL and complex comorbidities remains a significant clinical challenge.

There are significant regional disparities in the burden of AF/AFL correlated with metabolic risk factors. Deaths and DALYs linked to metabolic risk factors in AF/AFL were more prevalent in high-SDI regions than in low-SDI regions. However, a decreasing trend in the proportion of deaths and DALYs related to metabolic factors was observed in high-SDI regions, while low-SDI regions showed an increasing trend, reflecting an inverted V-shaped relationship between SDI and disease burden. At lower SDI levels, infectious and maternal-child conditions are predominant. As SDI rises, non-communicable diseases and injuries increase rapidly, often outpacing the capacity of health systems—resulting in a peak burden at middle SDI levels. In high-SDI regions, enhanced healthcare access and preventive measures contribute to a declining burden despite population aging. The mortality rate of AF/AFL is markedly lower in economically developed regions than in less developed ones, also highlighting persistent global inequities in healthcare access and resource distribution (9, 20–25). Moreover, economically disadvantaged regions are experiencing demographic aging alongside rising levels of certain metabolic and behavioral risks (26, 27). Consequently, the AF/AFL burden may increasingly shift toward populous, developing countries. These findings suggest that, despite previous efforts, strategies aimed at mitigating metabolic risk factors correlated with AF/AFL have yet to yield optimal results.

### 4.1 Limitation

The present study has several limitations, many of which have been acknowledged in previous GBD publications (1, 10, 11). First, there is significant heterogeneity in data quality across countries and regions. Although multiple statistical methods were used to adjust for these discrepancies, some degree of bias in the estimates remains inevitable. Additionally, the current GBD database lacks a systematic classification framework for AF subtypes and does not incorporate data on therapeutic interventions, such as anticoagulation therapy, ventricular rate control, and catheter ablation procedures. This analysis primarily focuses on the burden of AF/AFL associated with metabolic risk factors. However, it is important to note that the database does not comprehensively capture all relevant metabolic risk factors, and additional correlated factors may not have been included. Geographic disparities in healthcare resource allocation may compromise the accuracy of epidemiological assessments of AF/AFL disease burden. This challenge is particularly pronounced in regions with underdeveloped healthcare infrastructure, limited diagnostic capabilities, and suboptimal reporting mechanisms, which collectively hinder the validity and comprehensiveness of surveillance data.

## 5 Conclusions

In summary, our findings underscore the critical role of metabolic risk in AF/AFL-related mortality and DALYs, with a substantial increase observed over the past three decades. These trends vary significantly across regions, age groups, and sexes. Despite notable advances in the prevention and treatment of AF/AFL—particularly in high-SDI regions—the burden associated with metabolic risk continues to rise in low- and middle-SDI settings. Given the central role of modifiable metabolic risk factors in the prevention and management of AF/AFL, urgent action is needed to implement cost-effective strategies and interventions, especially in regions experiencing a high or growing disease burden.

## Data Availability

The original contributions presented in the study are included in the article/Supplementary Material, further inquiries can be directed to the corresponding authors.
