# Supplementary material for: Global, regional, and national burden of atrial fibrillation and flutter associated with metabolic risk factors, 1990–2021
Source: Front Cardiovasc Med. 2025 Sep 8;12:1578908. doi: 10.3389/fcvm.2025.1578908 (PMC12450952; doi:10.3389/fcvm.2025.1578908)
Supplement: Supplementary file 1 [file Datasheet1.docx]

Supplementary Material

# Supplementary Figures and Tables

## Supplementary Figures


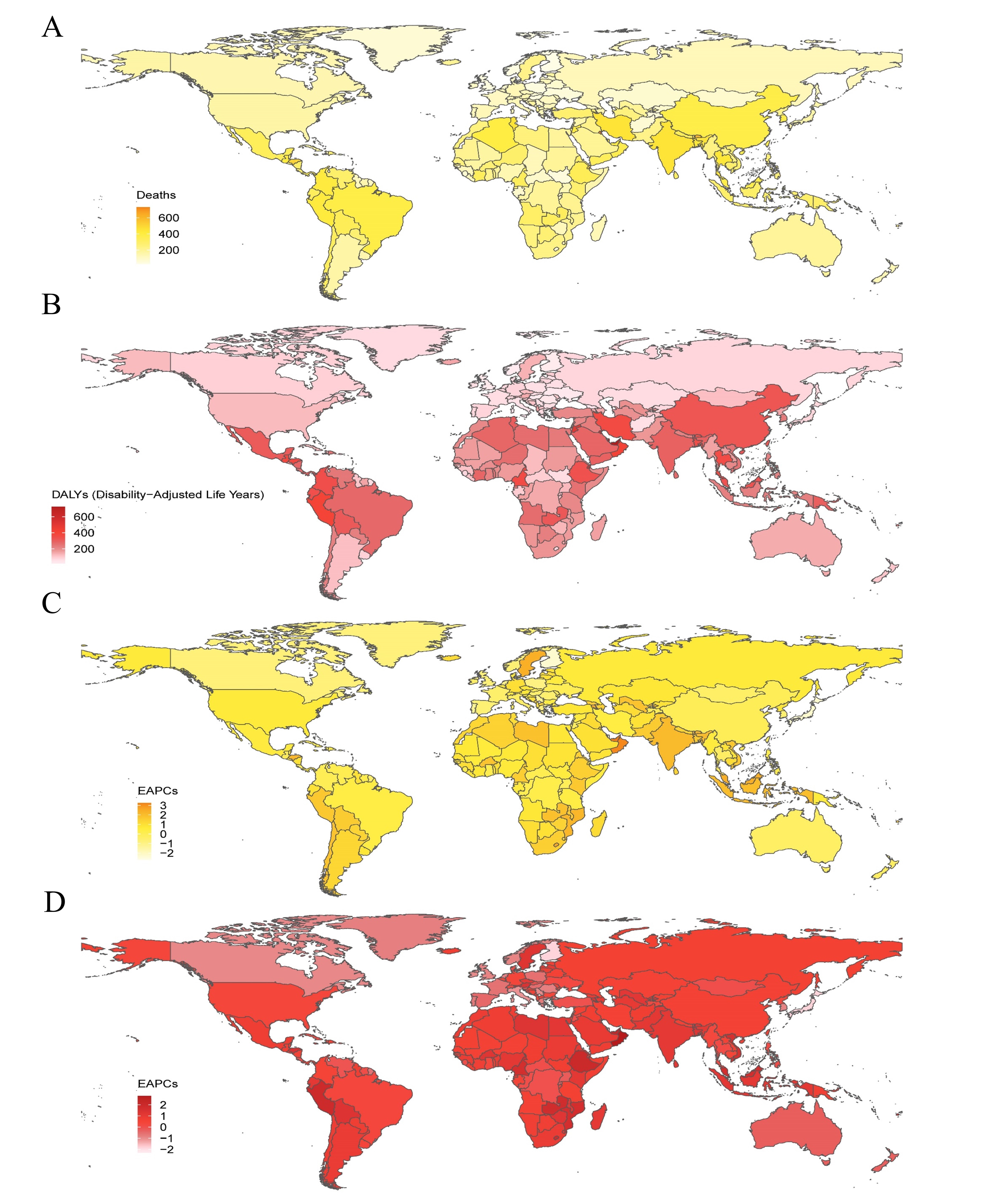


**Supplementary Figure S1**. The trend of global metabolic risks related atrial fibrillation/flutter burden in 204 countries and territories from 1990 to 2021. The absolute number changes of deaths (A) and disability-adjusted life years (B) changes compared to 1990. The estimated annual percentage changes of age-standardized death (C), and disability-adjusted life years (D) rates of atrial fibrillation/flutter across 204 countries and territories. DALYs, disability-adjusted life years; EAPCs, estimated annual percentage changes.

**
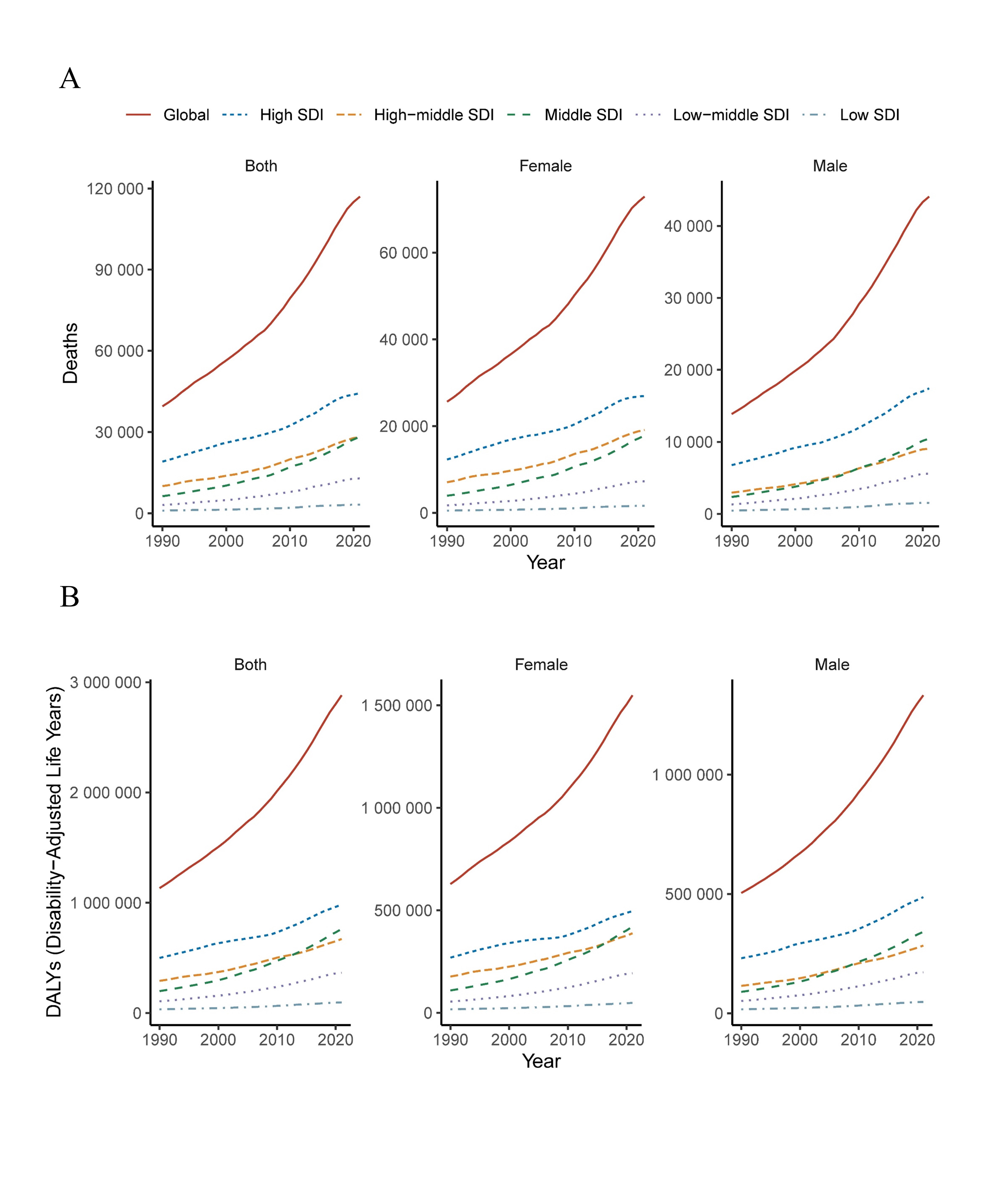
**

**Supplementary Figure S2**. Trends in number of deaths (A) and disability-adjusted life years (B) of atrial fibrillation/flutter related to metabolic risks in different Socio-Demographic Index regions from 1990–2021. DALYs, disability-adjusted life years; SDI, Socio-Demographic Index.

**
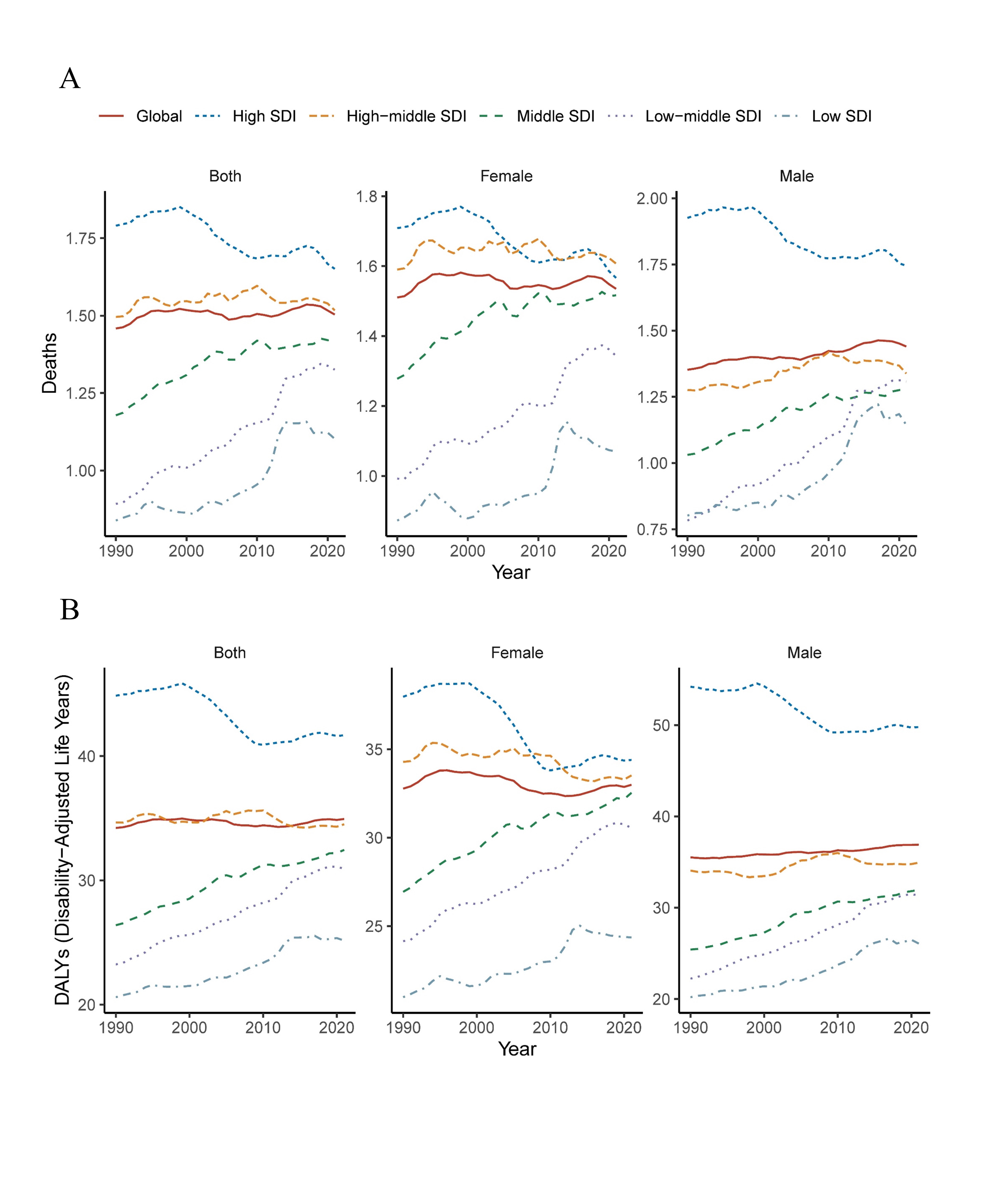
**

**Supplementary Figure S3**. Trends in age-standardized rates (per 100,000 persons) of deaths (A) and disability-adjusted life years (B) of atrial fibrillation/flutter related to metabolic risks at the global level in different Socio-Demographic Index regions from 1990 to 2021. DALYs, disability-adjusted life years; SDI, Socio-Demographic Index.


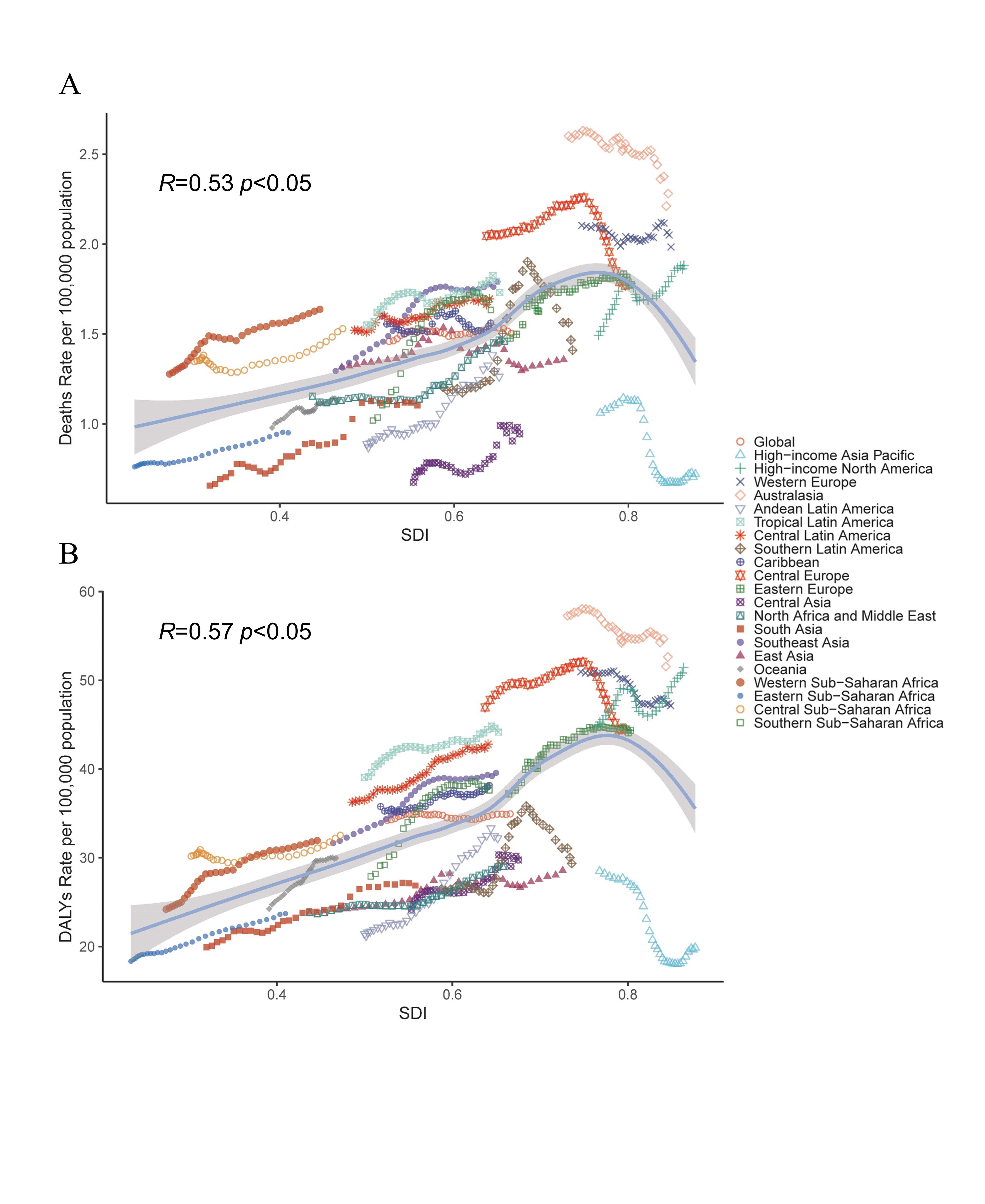


**Supplementary Figure S4.** Trends in age-standardized deaths and disability-adjusted life years associated with atrial fibrillation and flutter by socio-demographic index across 21 GBD regions, 1990–2021. Trends in age-standardized deaths (A) and DALYs (B) associated with AF/AFL, shown globally and across the 21 GBD regions, stratified by SDI, from 1990 to 2021. Each point represents an annual estimate, with values progressing from left to right by calendar year. DALYs, disability-adjusted life years; SDI, Socio-Demographic Index.

**
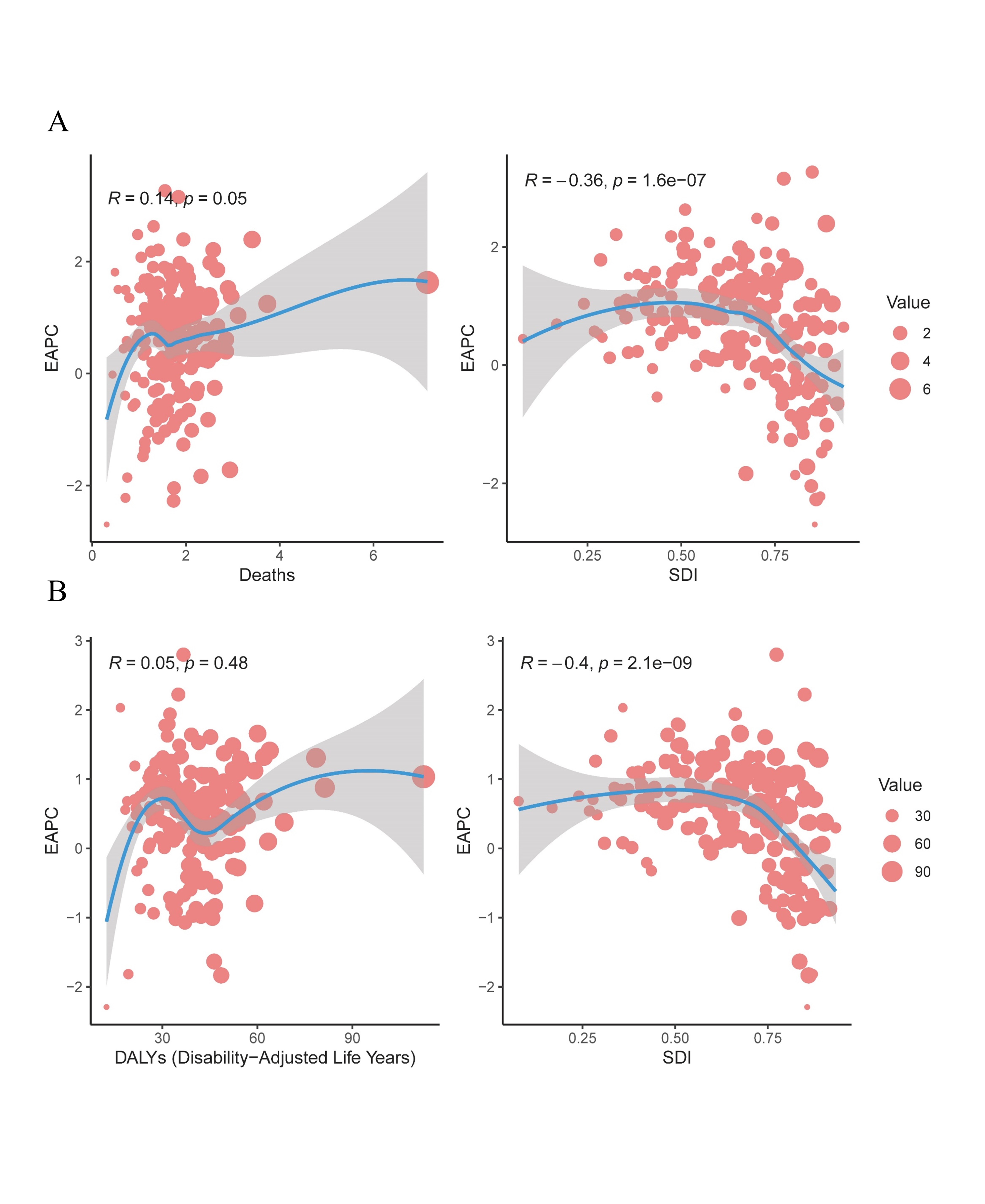
**

**Supplementary Figure S5**. The correlation between the estimated annual percentage changes of deaths/disability-adjusted life years with the rate of deaths/disability-adjusted life years and the corresponding Socio-Demographic Index in 2021. EAPC, estimated annual percentage change; DALYs, disability-adjusted life years; SDI, Socio-Demographic Index.

**
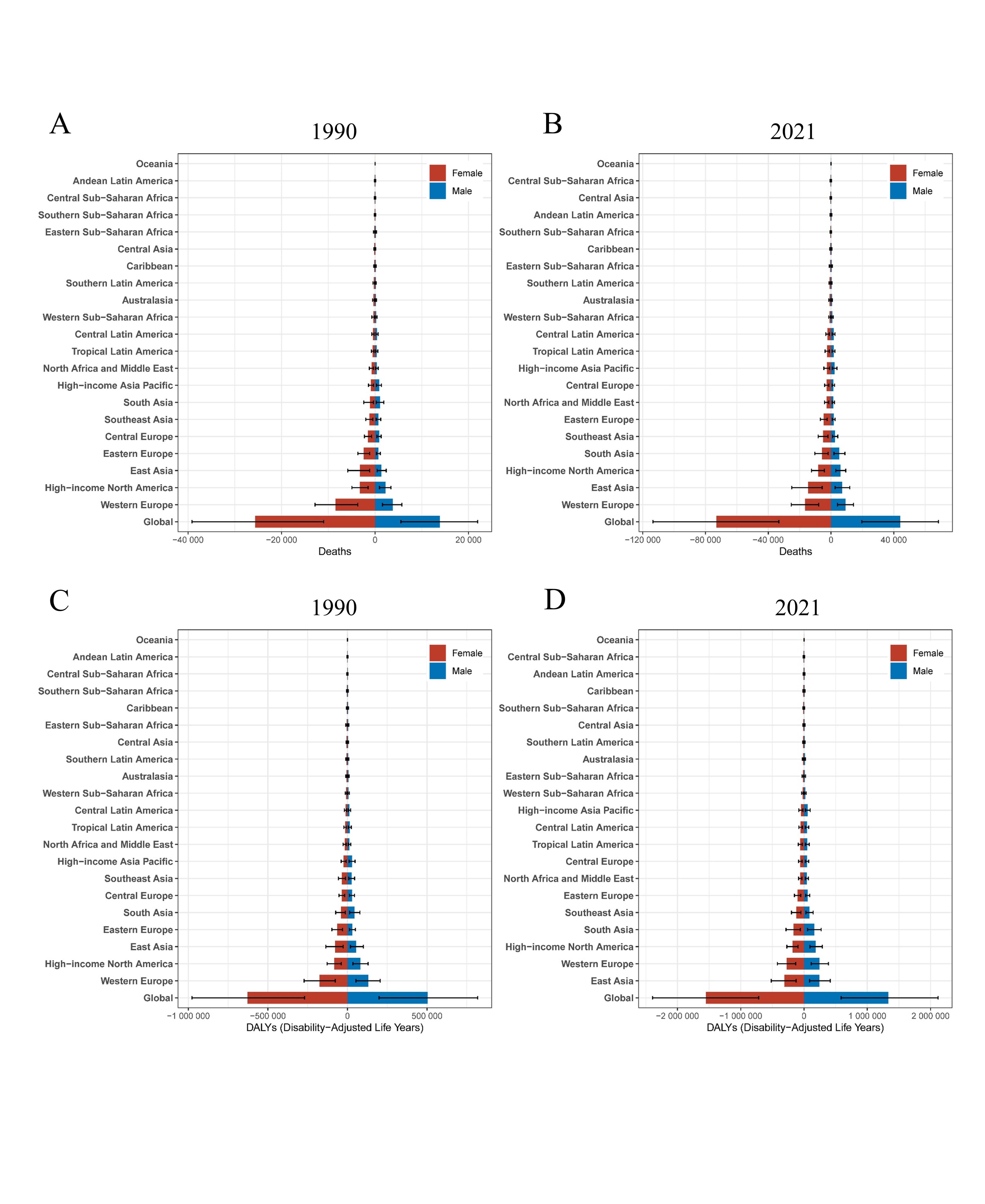
**

**Supplementary Figure S6.** Sex and region heterogeneities in atrial fibrillation/flutter burden related to metabolic risks in 1990 and 2019. Absolute number of deaths (A-B) and disability-adjusted life years (C-D) from metabolic risks related AF/AFL by sex in 21 regions in 1990 and 2021. DALYs, disability-adjusted life years.

**
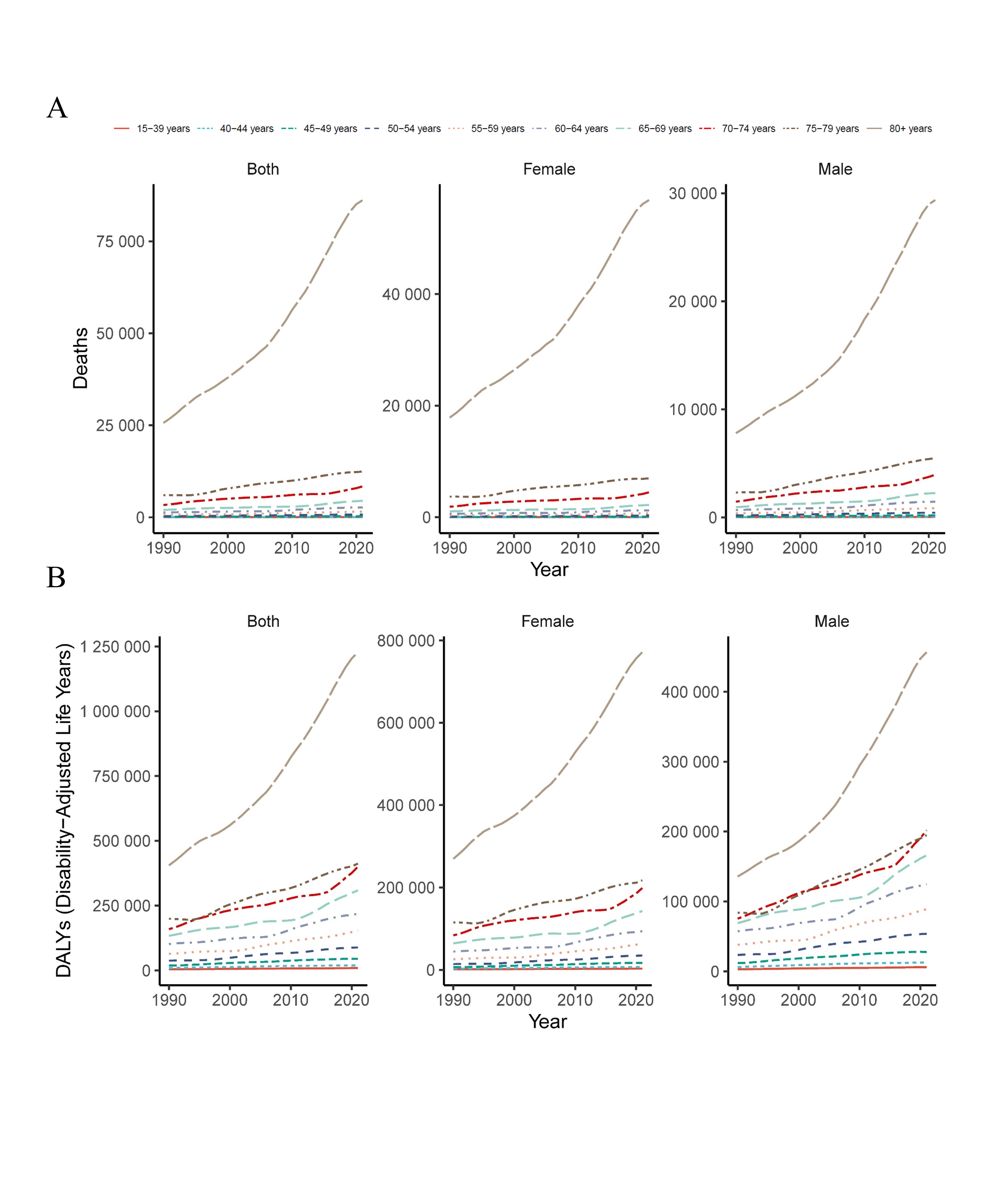
**

**Supplementary Figure S7**. Trends in numbers of deaths (A), and disability-adjusted life years disability-adjusted life years cases (B) of atrial fibrillation/flutter at the global level in different age group from 1990–2021.

**
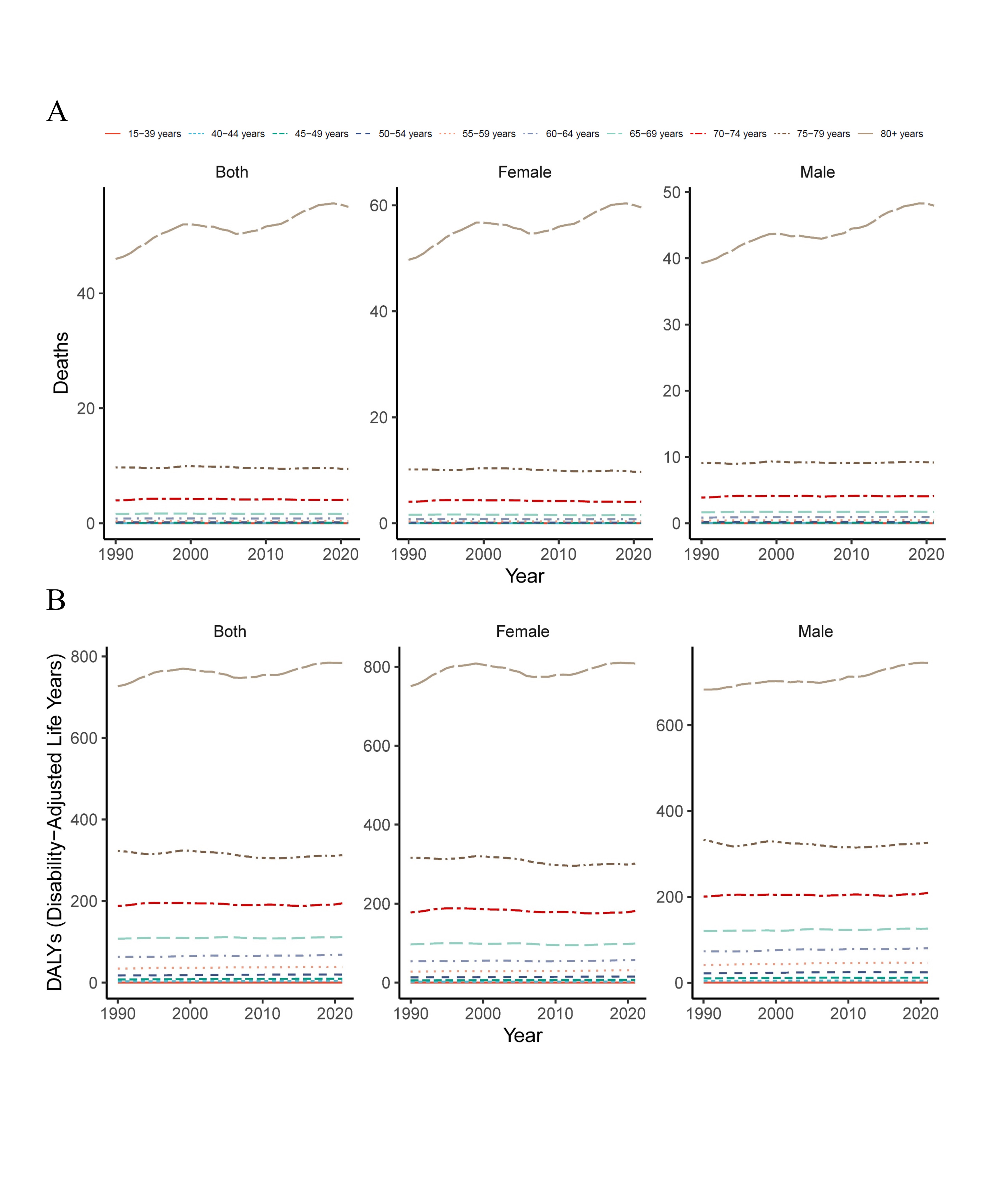
**

**Supplementary Figure S8**. Trends in rate of deaths (A), and disability-adjusted life years cases (B) of atrial fibrillation/flutter at the global level in different age group from 1990–2021.

**
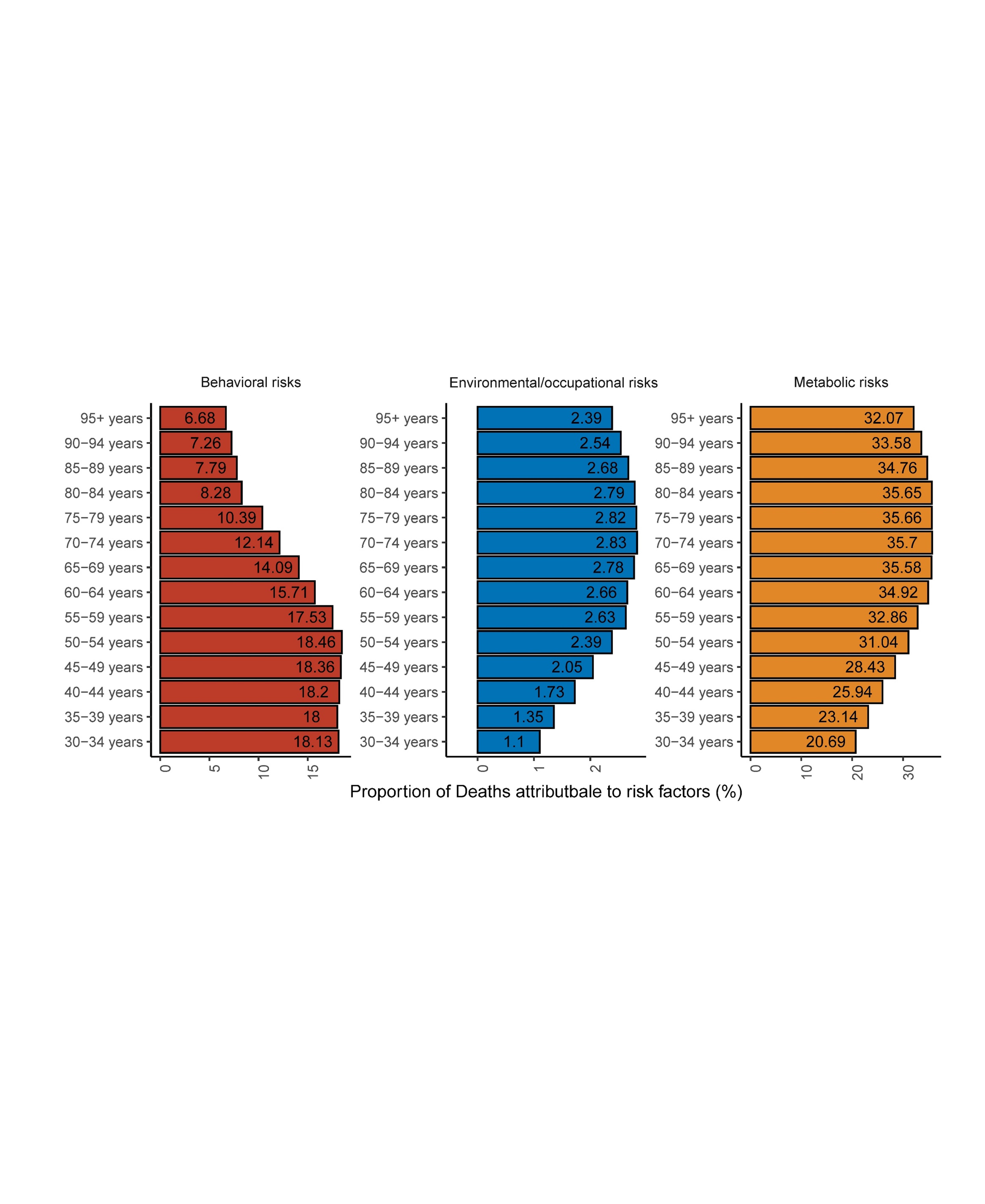
**

**Supplementary Figure S9**. The percentage contributions of major risk factors to death of atrial fibrillation/flutter in different age groups.

## Supplementary Tables

**Supplemental Table S1** Definitions of metabolic risk factors according to Global burden of disease

| **Factor** | **Definition** |
| --- | --- |
| High fasting plasma glucose | Serum fasting plasma glucose greater than 4.8-5.4 mmol/L |
| High LDL cholesterol | Low-density lipoprotein cholesterol greater than 0.7-1.3 mmol/L |
| High systolic blood pressure | Systolic blood pressure higher than 110-115 mmHg |
| High body mass index | Body mass index higher than 20-25 kg/m^2^ |
| Low bone mineral density | Bone mineral density measured by dual-X-ray-absorptiometry at the femoral neck (presented in g/cm^2^) lower than the 99th percentile of a reference population by age and sex |
| Kidney dysfunction | Estimated glomerular filtration rate (eGFR) less than 60 ml/min/1.73m^2^ or albumin to creatinine ratio (ACR) greater than or equal to 30 mg/g |

**Supplemental Table S2** Numbers and age standardized deaths rates of atrial fibrillation/flutter related to metabolic risks in 1990 and 2021, and their percentage change of death numbers, estimated annual percentage changes in age standardized death rate from 1990 to 2021, by Global Burden of Disease region and 204 countries

|  | 1990 | | 2021 | |  | |  |
| --- | --- | --- | --- | --- | --- | --- | --- |
|  | **Number of deaths (95% UI)** | **ASDR per 100000 people (95% UI)** | **Number of deaths (95% UI)** | **ASDR per 100000 people (95% UI)** | **PC of death numbers 1990-2021 (95% UI)** | **EAPC of ASDR 1990-2021 (95% CI)** | |
| **Regions** |  |  |  |  |  |  | |
| Andean Latin America | 144.29(57.84-249.47) | 0.89(0.35-1.54) | 688.42(311.49-1089.07) | 1.26(0.57-2.00) | 377.12(261.03-574.32) | 1.44(1.27-1.61) | |
| Australasia | 539.89(229.30-828.85) | 2.60(1.10-4.01) | 1494.83(713.78-2310.21) | 2.28(1.10-3.50) | 176.88(138.33-243.04) | -0.35(-0.44--0.26) | |
| Caribbean | 318.50(126.57-504.75) | 1.60(0.64-2.54) | 874.69(394.26-1371.31) | 1.56(0.71-2.44) | 174.63(133.24-244.28) | -0.01(-0.11-0.09) | |
| Central Asia | 251.22(113.93-384.95) | 0.68(0.31-1.04) | 555.71(268.56-821.07) | 0.95(0.45-1.40) | 121.20(85.87-159.31) | 1.01(0.75-1.28) | |
| Central Europe | 2410.40(1115.67-3545.52) | 2.05(0.94-3.01) | 4330.90(2158.76-6413.18) | 1.77(0.88-2.62) | 79.68(62.60-107.41) | -0.32(-0.56--0.07) | |
| Central Latin America | 867.14(368.52-1344.67) | 1.52(0.66-2.38) | 3849.96(1855.10-5731.87) | 1.70(0.82-2.52) | 343.98(288.27-432.78) | 0.39(0.33-0.44) | |
| Central Sub-Saharan Africa | 146.19(47.67-266.16) | 1.35(0.42-2.47) | 435.63(148.06-785.25) | 1.53(0.51-2.82) | 197.99(123.64-298.35) | 0.27(0.13-0.42) | |
| East Asia | 4580.46(1618.04-7891.10) | 1.32(0.45-2.38) | 21634.45(8624.31-35403.59) | 1.36(0.54-2.27) | 372.32(232.92-614.37) | -0.06(-0.24-0.13) | |
| Eastern Europe | 3152.12(1457.61-4837.29) | 1.48(0.68-2.28) | 6433.93(3230.05-9306.18) | 1.78(0.89-2.57) | 104.11(76.60-142.80) | 0.50(0.39-0.62) | |
| Eastern Sub-Saharan Africa | 305.83(93.64-584.36) | 0.76(0.24-1.49) | 942.95(307.01-1740.47) | 0.95(0.31-1.76) | 208.33(142.68-298.24) | 0.73(0.64-0.83) | |
| High-income Asia Pacific | 1756.19(649.25-2768.58) | 1.06(0.39-1.69) | 4933.80(1787.14-8208.74) | 0.72(0.27-1.20) | 180.94(127.24-227.65) | -2.05(-2.47--1.62) | |
| High-income North America | 5494.22(2461.34-8386.59) | 1.49(0.67-2.28) | 14310.28(7170.01-21652.64) | 1.88(0.95-2.85) | 160.46(125.52-219.94) | 0.52(0.37-0.66) | |
| North Africa and Middle East | 1129.74(483.18-1809.87) | 1.15(0.49-1.86) | 4235.17(2191.15-6268.11) | 1.46(0.74-2.17) | 274.88(184.66-416.57) | 0.87(0.69-1.05) | |
| Oceania | 14.32(5.57-24.44) | 0.98(0.38-1.68) | 50.25(22.01-83.06) | 1.14(0.48-1.88) | 250.77(192.38-327.97) | 0.37(0.30-0.44) | |
| South Asia | 2128.03(614.46-4075.55) | 0.66(0.19-1.27) | 10994.60(3931.94-18897.75) | 1.10(0.39-1.91) | 416.66(281.75-616.97) | 1.85(1.64-2.06) | |
| Southeast Asia | 1888.96(702.77-3105.04) | 1.29(0.48-2.13) | 7671.95(2991.51-12160.82) | 1.79(0.69-2.85) | 306.15(217.28-428.04) | 1.05(0.89-1.20) | |
| Southern Latin America | 437.23(190.09-672.62) | 1.20(0.52-1.88) | 1319.52(633.04-1967.75) | 1.41(0.68-2.10) | 201.79(160.41-264.81) | 1.43(0.98-1.88) | |
| Southern Sub-Saharan Africa | 186.40(81.29-294.24) | 1.02(0.44-1.63) | 585.11(287.16-853.49) | 1.63(0.79-2.43) | 213.90(142.08-315.96) | 1.53(1.18-1.88) | |
| Tropical Latin America | 860.94(358.19-1353.99) | 1.55(0.63-2.47) | 4114.98(1888.31-6294.56) | 1.73(0.79-2.65) | 377.96(322.51-485.81) | 0.34(0.25-0.43) | |
| Western Europe | 12293.49(5405.10-18648.85) | 2.10(0.92-3.20) | 25859.45(11813.39-39983.81) | 1.98(0.92-3.06) | 110.35(87.71-137.65) | -0.04(-0.11-0.03) | |
| Western Sub-Saharan Africa | 615.30(239.46-1046.12) | 1.28(0.50-2.17) | 1695.91(726.92-2606.40) | 1.64(0.71-2.56) | 175.62(111.06-283.82) | 0.78(0.71-0.84) | |
| **Countries** |  |  |  |  |  |  | |
| Afghanistan | 33.46(9.34-69.02) | 0.79(0.21-1.62) | 57.00(21.11-100.27) | 1.02(0.36-1.80) | 70.35(23.49-148.53) | 0.95(0.86-1.04) | |
| Albania | 22.90(10.17-35.27) | 1.55(0.69-2.39) | 73.20(33.80-112.94) | 1.89(0.87-2.96) | 219.65(132.64-329.71) | 1.03(0.84-1.23) | |
| Algeria | 87.90(33.59-148.83) | 1.84(0.67-3.12) | 432.62(208.72-692.66) | 2.28(1.09-3.64) | 392.16(239.32-649.15) | 1.43(1.16-1.69) | |
| American Samoa | 0.22(0.10-0.36) | 1.67(0.75-2.92) | 0.82(0.41-1.33) | 2.50(1.20-4.11) | 279.27(189.05-401.94) | 1.40(1.29-1.52) | |
| Andorra | 0.70(0.27-1.26) | 1.73(0.67-3.07) | 2.44(1.02-4.06) | 1.30(0.55-2.14) | 246.59(117.16-441.86) | -0.66(-0.84--0.47) | |
| Angola | 23.42(7.78-43.38) | 1.21(0.38-2.22) | 94.13(32.74-159.25) | 1.61(0.55-2.82) | 301.84(174.67-485.73) | 0.77(0.68-0.87) | |
| Antigua and Barbuda | 1.24(0.50-2.03) | 2.09(0.85-3.40) | 1.73(0.82-2.70) | 2.18(1.01-3.42) | 38.90(9.69-90.89) | 0.31(0.09-0.54) | |
| Argentina | 293.85(126.13-465.69) | 1.18(0.50-1.88) | 779.33(364.38-1195.05) | 1.31(0.61-2.01) | 165.21(117.20-237.39) | 1.29(0.85-1.73) | |
| Armenia | 10.70(4.85-16.41) | 0.52(0.23-0.81) | 41.58(19.23-63.94) | 0.97(0.45-1.49) | 288.57(203.22-401.39) | 2.48(2.09-2.88) | |
| Australia | 431.08(181.74-664.15) | 2.51(1.05-3.87) | 1242.11(600.80-1930.33) | 2.23(1.08-3.43) | 188.14(141.53-265.62) | -0.36(-0.48--0.24) | |
| Austria | 243.26(103.87-370.58) | 2.02(0.88-3.11) | 592.22(271.50-944.86) | 2.43(1.12-3.84) | 143.46(99.62-195.11) | 0.71(0.40-1.03) | |
| Azerbaijan | 19.59(8.20-29.98) | 0.54(0.22-0.85) | 49.39(22.85-76.62) | 0.71(0.33-1.12) | 152.09(75.41-256.57) | 1.49(1.21-1.77) | |
| Bahamas | 2.10(0.85-3.30) | 1.77(0.72-2.82) | 6.57(3.08-10.23) | 2.11(0.98-3.31) | 212.38(135.38-328.87) | 0.55(0.31-0.78) | |
| Bahrain | 1.30(0.45-2.25) | 2.37(0.77-4.29) | 6.00(2.47-10.28) | 2.24(0.79-3.92) | 361.58(225.62-578.50) | -0.27(-0.70-0.17) | |
| Bangladesh | 257.42(66.87-504.38) | 0.80(0.21-1.62) | 1354.63(436.82-2365.87) | 1.40(0.46-2.47) | 426.23(264.84-760.34) | 1.63(1.41-1.85) | |
| Barbados | 4.95(1.93-7.84) | 1.67(0.65-2.68) | 11.03(5.12-17.81) | 2.15(1.00-3.48) | 122.90(76.11-197.25) | 1.00(0.75-1.26) | |
| Belarus | 156.65(67.05-240.80) | 1.36(0.58-2.10) | 279.45(135.88-421.70) | 1.68(0.82-2.53) | 78.39(35.89-136.46) | 0.50(0.41-0.58) | |
| Belgium | 232.89(95.35-375.29) | 1.51(0.62-2.45) | 521.00(231.79-803.61) | 1.58(0.70-2.43) | 123.71(83.19-182.81) | 0.60(0.33-0.88) | |
| Belize | 1.10(0.47-1.78) | 1.22(0.52-1.97) | 3.90(1.92-5.99) | 1.65(0.81-2.53) | 255.15(161.93-398.36) | 0.79(0.28-1.30) | |
| Benin | 12.87(4.26-22.39) | 0.93(0.31-1.65) | 37.26(14.90-61.45) | 1.25(0.48-2.06) | 189.57(108.46-312.13) | 1.06(0.99-1.13) | |
| Bermuda | 1.00(0.42-1.62) | 2.03(0.83-3.27) | 2.58(1.21-4.06) | 1.55(0.73-2.45) | 157.40(93.67-264.58) | -1.03(-1.11--0.94) | |
| Bhutan | 0.89(0.26-1.84) | 0.69(0.19-1.50) | 6.20(2.07-11.14) | 1.26(0.42-2.28) | 599.86(353.74-1005.03) | 2.18(2.08-2.28) | |
| Bolivia (Plurinational State of) | 24.02(7.81-44.86) | 1.16(0.37-2.19) | 106.85(44.30-182.60) | 1.70(0.69-2.86) | 344.79(209.86-582.02) | 1.61(1.46-1.76) | |
| Bosnia and Herzegovina | 36.20(14.89-55.31) | 1.26(0.51-1.96) | 102.75(48.13-155.21) | 1.61(0.75-2.44) | 183.84(105.38-303.43) | 0.95(0.85-1.05) | |
| Botswana | 2.76(1.02-4.82) | 1.02(0.37-1.81) | 10.13(4.68-16.40) | 1.24(0.56-2.04) | 266.82(137.14-531.20) | 0.99(0.72-1.26) | |
| Brazil | 832.96(346.70-1309.55) | 1.55(0.63-2.47) | 4010.04(1834.75-6128.53) | 1.72(0.79-2.64) | 381.42(325.18-492.06) | 0.31(0.22-0.41) | |
| Brunei Darussalam | 1.63(0.55-2.77) | 2.32(0.77-4.08) | 3.59(1.38-5.92) | 1.83(0.65-3.15) | 120.21(60.97-236.14) | -0.21(-0.49-0.07) | |
| Bulgaria | 162.44(77.27-237.63) | 2.25(1.06-3.31) | 364.10(172.85-543.54) | 2.46(1.16-3.68) | 124.14(80.94-174.05) | 0.40(0.23-0.57) | |
| Burkina Faso | 23.83(7.38-43.31) | 1.08(0.33-1.97) | 87.14(29.40-146.96) | 1.66(0.58-2.82) | 265.67(161.47-411.60) | 1.78(1.61-1.96) | |
| Burundi | 9.99(2.33-22.99) | 0.66(0.15-1.63) | 21.63(5.60-48.65) | 0.84(0.21-1.90) | 116.67(42.38-236.23) | 0.47(0.34-0.59) | |
| Cabo Verde | 2.62(0.96-4.67) | 1.11(0.41-1.99) | 8.32(3.25-13.84) | 2.02(0.79-3.35) | 217.60(73.81-415.70) | 1.85(1.62-2.09) | |
| Cambodia | 20.53(6.77-36.37) | 0.90(0.29-1.62) | 79.16(26.40-137.61) | 1.27(0.42-2.20) | 285.68(161.86-435.97) | 1.16(1.00-1.32) | |
| Cameroon | 30.29(10.46-52.05) | 1.27(0.43-2.21) | 130.88(56.12-199.84) | 2.07(0.87-3.12) | 332.11(212.06-560.53) | 1.55(1.28-1.83) | |
| Canada | 536.46(234.81-825.08) | 1.73(0.76-2.68) | 1317.35(648.25-2015.03) | 1.49(0.73-2.27) | 145.56(107.74-199.80) | -0.77(-0.91--0.62) | |
| Central African Republic | 7.07(2.09-13.75) | 1.30(0.38-2.57) | 13.45(4.08-25.71) | 1.36(0.40-2.71) | 90.28(39.91-157.73) | 0.12(0.09-0.15) | |
| Chad | 15.52(5.07-30.10) | 0.85(0.27-1.67) | 34.80(12.30-59.58) | 1.16(0.39-1.97) | 124.31(56.06-228.91) | 1.04(1.00-1.08) | |
| Chile | 101.30(45.36-151.42) | 1.31(0.58-1.97) | 436.15(206.27-634.94) | 1.63(0.77-2.37) | 330.55(280.60-406.60) | 1.86(1.29-2.44) | |
| China | 4337.72(1520.50-7531.57) | 1.31(0.45-2.38) | 20752.99(8276.98-34233.63) | 1.37(0.54-2.30) | 378.43(232.20-632.56) | -0.01(-0.20-0.17) | |
| Colombia | 185.44(76.15-290.34) | 1.50(0.61-2.36) | 925.24(409.92-1397.37) | 1.61(0.71-2.43) | 398.94(300.63-549.94) | 0.02(-0.09-0.13) | |
| Comoros | 0.99(0.28-1.95) | 0.90(0.25-1.82) | 3.56(1.16-6.99) | 1.12(0.37-2.24) | 259.96(143.26-446.30) | 0.58(0.51-0.66) | |
| Congo | 10.82(3.69-19.06) | 1.95(0.65-3.47) | 29.08(11.38-47.21) | 2.10(0.78-3.42) | 168.84(92.18-284.84) | 0.10(-0.01-0.21) | |
| Cook Islands | 0.15(0.07-0.25) | 1.77(0.80-2.97) | 0.53(0.26-0.86) | 2.25(1.11-3.66) | 251.93(150.97-384.16) | 0.75(0.66-0.83) | |
| Costa Rica | 23.87(9.96-37.87) | 1.57(0.65-2.50) | 96.44(44.73-148.72) | 1.67(0.78-2.55) | 304.03(232.40-415.55) | -0.02(-0.23-0.20) | |
| Croatia | 59.45(27.20-88.61) | 1.26(0.57-1.87) | 148.05(71.30-221.65) | 1.43(0.69-2.14) | 149.04(108.55-203.39) | 0.58(0.13-1.03) | |
| Cuba | 121.78(42.63-204.97) | 1.44(0.50-2.44) | 334.89(153.26-549.74) | 1.50(0.69-2.46) | 174.99(111.62-285.21) | 0.16(0.03-0.30) | |
| Cyprus | 20.73(7.65-35.44) | 4.85(1.76-8.29) | 44.24(18.95-71.37) | 2.94(1.24-4.80) | 113.43(44.25-242.32) | -1.72(-2.02--1.42) | |
| Czechia | 199.46(94.75-297.23) | 1.61(0.76-2.39) | 445.37(221.89-672.31) | 1.86(0.93-2.80) | 123.29(89.88-166.96) | 0.77(0.64-0.89) | |
| Côte d'Ivoire | 20.69(7.35-35.05) | 1.32(0.46-2.23) | 89.01(34.52-138.88) | 1.70(0.67-2.70) | 330.22(212.94-529.59) | 0.77(0.67-0.88) | |
| Democratic People's Republic of Korea | 117.71(38.44-214.09) | 1.39(0.44-2.66) | 370.76(135.30-645.87) | 1.60(0.56-2.83) | 214.96(120.70-388.24) | 0.77(0.61-0.93) | |
| Democratic Republic of the Congo | 94.95(29.89-179.20) | 1.29(0.38-2.46) | 277.51(88.47-532.84) | 1.43(0.44-2.79) | 192.26(107.01-318.07) | 0.23(0.06-0.40) | |
| Denmark | 163.06(67.71-255.08) | 1.84(0.76-2.87) | 319.37(132.55-503.31) | 2.22(0.93-3.48) | 95.86(65.38-127.59) | 0.64(0.18-1.09) | |
| Djibouti | 0.57(0.18-1.02) | 0.96(0.30-1.79) | 3.43(1.15-6.34) | 1.24(0.41-2.27) | 504.52(313.94-797.56) | 0.80(0.77-0.84) | |
| Dominica | 1.31(0.61-2.09) | 2.52(1.16-3.95) | 2.00(1.04-3.07) | 2.85(1.48-4.34) | 52.91(13.64-113.02) | 0.39(0.31-0.48) | |
| Dominican Republic | 37.96(13.44-62.84) | 1.68(0.59-2.79) | 141.17(58.33-232.64) | 1.50(0.62-2.47) | 271.85(161.09-467.71) | 0.18(-0.18-0.55) | |
| Ecuador | 41.48(16.19-70.42) | 1.08(0.40-1.85) | 178.86(89.80-281.31) | 1.31(0.65-2.06) | 331.14(221.72-568.91) | 0.98(0.81-1.16) | |
| Egypt | 140.71(64.42-235.06) | 1.22(0.55-2.10) | 400.74(222.59-586.25) | 1.42(0.76-2.11) | 184.79(106.37-313.18) | 0.81(0.68-0.94) | |
| El Salvador | 41.55(16.80-68.14) | 1.51(0.61-2.49) | 146.41(69.42-235.03) | 1.98(0.94-3.14) | 252.39(154.01-398.71) | 0.91(0.79-1.02) | |
| Equatorial Guinea | 1.48(0.50-2.85) | 1.37(0.47-2.63) | 5.72(2.37-9.54) | 2.04(0.83-3.37) | 285.01(131.35-595.26) | 1.36(1.27-1.45) | |
| Eritrea | 3.65(1.08-7.21) | 0.79(0.24-1.61) | 14.80(4.29-30.83) | 1.24(0.35-2.65) | 305.48(156.34-532.38) | 1.47(1.40-1.54) | |
| Estonia | 27.78(12.58-42.20) | 1.54(0.69-2.32) | 77.48(37.79-116.92) | 2.19(1.07-3.28) | 178.87(128.76-245.87) | 1.02(0.90-1.14) | |
| Eswatini | 2.02(0.89-3.39) | 1.33(0.59-2.20) | 4.64(2.25-7.23) | 1.60(0.78-2.43) | 129.56(51.86-259.12) | 1.22(0.85-1.60) | |
| Ethiopia | 35.92(9.23-80.61) | 0.36(0.09-0.79) | 159.37(45.95-321.03) | 0.55(0.15-1.12) | 343.72(190.32-596.28) | 1.50(1.39-1.62) | |
| Fiji | 2.82(1.31-4.46) | 1.39(0.62-2.28) | 10.95(5.41-16.62) | 2.67(1.30-4.02) | 287.66(181.37-438.42) | 1.85(1.50-2.20) | |
| Finland | 231.00(99.00-342.94) | 3.25(1.39-4.83) | 292.06(134.11-437.06) | 1.74(0.81-2.62) | 26.43(4.94-56.23) | -2.27(-2.52--2.02) | |
| France | 1709.51(691.74-2675.02) | 1.89(0.76-2.94) | 3497.65(1521.15-5494.68) | 1.63(0.72-2.54) | 104.60(67.23-156.37) | -0.48(-0.57--0.38) | |
| Gabon | 8.45(3.10-15.04) | 2.13(0.77-3.81) | 15.75(6.68-24.96) | 2.60(1.11-4.24) | 86.51(24.53-169.47) | 0.47(0.34-0.59) | |
| Gambia | 2.16(0.74-3.75) | 1.25(0.45-2.19) | 10.70(4.20-17.60) | 1.83(0.70-3.10) | 396.39(231.72-673.13) | 1.24(1.18-1.29) | |
| Georgia | 64.64(28.09-105.73) | 1.22(0.53-2.04) | 135.77(62.32-204.43) | 2.02(0.93-3.03) | 110.06(57.30-195.37) | 1.62(0.66-2.58) | |
| Germany | 3513.88(1553.13-5247.74) | 2.68(1.18-4.03) | 8231.63(3838.44-12472.58) | 3.11(1.46-4.72) | 134.26(98.74-179.74) | 1.04(0.80-1.27) | |
| Ghana | 42.37(15.32-72.36) | 1.41(0.50-2.42) | 132.79(55.37-212.84) | 1.62(0.67-2.63) | 213.39(115.77-355.91) | 0.15(-0.03-0.33) | |
| Greece | 217.65(89.19-329.34) | 1.63(0.66-2.50) | 483.29(215.86-770.45) | 1.36(0.62-2.15) | 122.05(85.43-179.59) | -0.85(-0.97--0.73) | |
| Greenland | 0.62(0.28-0.93) | 3.41(1.51-5.31) | 1.05(0.52-1.67) | 2.47(1.17-3.91) | 70.33(34.53-125.90) | -0.82(-0.96--0.69) | |
| Grenada | 1.38(0.49-2.28) | 1.56(0.56-2.60) | 1.79(0.80-2.81) | 2.28(0.98-3.60) | 30.27(-1.28-87.77) | 1.21(0.84-1.58) | |
| Guam | 0.62(0.28-1.02) | 1.79(0.69-3.00) | 1.74(0.79-2.77) | 0.75(0.35-1.17) | 179.34(111.17-268.67) | -1.86(-2.33--1.38) | |
| Guatemala | 21.85(8.97-34.69) | 1.33(0.54-2.23) | 112.01(51.60-180.03) | 1.31(0.60-2.09) | 412.66(297.50-586.94) | -0.01(-0.18-0.16) | |
| Guinea | 20.98(6.30-40.44) | 0.98(0.29-1.91) | 47.07(17.02-78.64) | 1.32(0.47-2.23) | 124.31(54.88-241.72) | 1.06(1.01-1.10) | |
| Guinea-Bissau | 2.39(0.84-4.24) | 1.29(0.47-2.33) | 5.04(1.87-8.59) | 1.75(0.64-3.05) | 111.01(48.30-204.40) | 1.09(1.03-1.15) | |
| Guyana | 3.68(1.50-6.04) | 1.39(0.56-2.31) | 7.88(3.46-12.83) | 1.80(0.80-2.95) | 113.97(59.41-189.42) | 0.96(0.60-1.32) | |
| Haiti | 27.46(8.67-53.25) | 1.60(0.51-3.11) | 69.21(22.14-124.65) | 1.69(0.55-3.09) | 152.05(75.03-277.00) | 0.36(0.30-0.42) | |
| Honduras | 17.42(6.66-29.74) | 1.31(0.49-2.31) | 106.35(46.27-175.06) | 2.58(1.09-4.21) | 510.54(283.52-833.79) | 2.21(1.85-2.57) | |
| Hungary | 246.60(115.51-351.30) | 2.01(0.94-2.87) | 362.49(184.24-519.87) | 1.62(0.82-2.32) | 46.99(25.97-76.57) | -0.48(-0.62--0.34) | |
| Iceland | 6.09(2.64-9.91) | 1.93(0.84-3.13) | 18.03(8.34-28.00) | 2.44(1.13-3.75) | 196.07(139.86-267.35) | 1.17(0.97-1.37) | |
| India | 1533.04(456.17-2951.99) | 0.61(0.18-1.16) | 8513.13(2971.25-14487.16) | 1.04(0.36-1.78) | 455.31(297.97-676.49) | 2.08(1.78-2.37) | |
| Indonesia | 709.27(244.57-1190.64) | 1.37(0.45-2.32) | 2775.33(1066.96-4455.11) | 2.52(0.97-4.06) | 291.30(185.77-430.87) | 1.98(1.85-2.11) | |
| Iran (Islamic Republic of) | 123.37(50.04-203.40) | 0.98(0.39-1.64) | 681.10(337.92-1056.71) | 1.14(0.56-1.78) | 452.08(303.38-705.07) | 0.41(0.26-0.55) | |
| Iraq | 84.69(37.50-138.36) | 1.22(0.54-2.00) | 271.54(129.70-437.06) | 2.01(0.94-3.28) | 220.62(119.69-420.27) | 1.18(0.96-1.39) | |
| Ireland | 84.51(34.83-128.30) | 2.29(0.93-3.49) | 171.78(74.01-270.10) | 1.96(0.85-3.08) | 103.27(68.08-145.77) | -0.35(-0.63--0.07) | |
| Israel | 97.82(42.39-146.47) | 2.33(1.00-3.49) | 276.32(127.38-418.32) | 1.88(0.87-2.83) | 182.48(139.83-230.24) | -0.41(-0.64--0.18) | |
| Italy | 1315.71(531.53-2010.05) | 1.59(0.64-2.46) | 3040.22(1454.54-4758.81) | 1.41(0.67-2.20) | 131.07(83.43-194.73) | -0.28(-0.45--0.11) | |
| Jamaica | 26.59(10.35-43.75) | 1.44(0.56-2.35) | 67.08(29.29-109.94) | 1.82(0.81-2.97) | 152.33(86.08-257.18) | 0.82(0.63-1.02) | |
| Japan | 1579.92(594.07-2487.73) | 1.07(0.40-1.70) | 4113.63(1505.46-6785.77) | 0.71(0.26-1.15) | 160.37(110.36-204.64) | -2.22(-2.70--1.74) | |
| Jordan | 8.34(3.96-13.12) | 1.10(0.51-1.74) | 42.23(22.37-65.60) | 1.10(0.56-1.70) | 406.18(261.20-591.40) | -0.16(-0.43-0.11) | |
| Kazakhstan | 88.09(40.75-138.23) | 0.91(0.42-1.46) | 157.90(77.46-230.79) | 1.35(0.66-1.96) | 79.25(32.41-131.97) | 0.80(0.54-1.06) | |
| Kenya | 35.70(12.20-64.79) | 0.70(0.24-1.30) | 135.92(47.62-243.11) | 1.12(0.37-2.04) | 280.77(181.00-436.61) | 1.68(1.56-1.81) | |
| Kiribati | 0.16(0.07-0.27) | 0.71(0.28-1.32) | 0.45(0.21-0.71) | 1.23(0.52-2.02) | 181.64(96.73-320.77) | 1.61(1.44-1.78) | |
| Kuwait | 3.14(1.41-4.93) | 0.95(0.42-1.52) | 26.11(14.13-38.77) | 1.30(0.70-1.94) | 732.53(553.74-1054.69) | 1.37(0.76-1.98) | |
| Kyrgyzstan | 15.37(6.73-25.02) | 0.66(0.29-1.08) | 27.86(12.89-42.11) | 0.84(0.39-1.28) | 81.24(33.30-147.48) | 0.95(0.70-1.21) | |
| Lao People's Democratic Republic | 11.86(3.72-21.88) | 1.21(0.39-2.26) | 40.37(15.05-69.19) | 1.60(0.60-2.77) | 240.35(143.57-401.99) | 0.90(0.88-0.93) | |
| Latvia | 47.48(22.00-72.08) | 1.45(0.68-2.19) | 93.00(44.27-140.02) | 1.86(0.89-2.80) | 95.86(67.59-137.45) | 1.05(0.82-1.27) | |
| Lebanon | 22.75(8.35-40.38) | 1.52(0.56-2.73) | 83.42(40.53-128.24) | 1.20(0.58-1.83) | 266.61(118.78-621.36) | -1.04(-1.22--0.87) | |
| Lesotho | 4.51(1.81-8.59) | 0.79(0.31-1.57) | 8.06(3.75-13.14) | 1.31(0.62-2.09) | 78.65(-1.04-171.33) | 2.63(2.17-3.09) | |
| Liberia | 8.47(2.95-14.66) | 1.32(0.44-2.30) | 18.57(7.61-30.98) | 1.66(0.66-2.78) | 119.27(57.93-225.61) | 0.80(0.75-0.86) | |
| Libya | 13.56(5.87-22.77) | 0.89(0.38-1.49) | 47.44(20.30-83.81) | 1.30(0.56-2.30) | 249.74(113.86-488.95) | 1.90(1.63-2.18) | |
| Lithuania | 60.02(27.84-92.25) | 1.42(0.65-2.17) | 137.09(71.55-204.43) | 1.90(0.99-2.81) | 128.40(94.04-181.78) | 1.02(0.90-1.14) | |
| Luxembourg | 12.38(5.45-18.70) | 2.47(1.10-3.76) | 30.81(14.42-46.60) | 2.37(1.11-3.59) | 148.80(102.46-207.23) | 0.25(0.08-0.42) | |
| Madagascar | 36.52(10.91-68.37) | 1.14(0.35-2.19) | 85.87(27.56-156.05) | 1.62(0.52-2.99) | 135.11(67.69-245.90) | 1.19(1.13-1.25) | |
| Malawi | 13.54(3.49-25.24) | 0.63(0.16-1.22) | 43.00(14.53-75.79) | 1.02(0.34-1.79) | 217.64(136.67-394.68) | 1.53(1.44-1.63) | |
| Malaysia | 78.04(28.01-126.95) | 1.06(0.38-1.74) | 371.30(155.74-566.56) | 1.95(0.81-2.99) | 375.80(276.09-536.84) | 2.40(2.03-2.76) | |
| Maldives | 0.42(0.13-0.73) | 1.13(0.31-2.04) | 3.06(1.05-5.22) | 1.28(0.43-2.20) | 637.60(349.45-1095.25) | 0.20(0.03-0.37) | |
| Mali | 16.30(5.33-30.70) | 0.92(0.29-1.70) | 44.65(15.38-74.80) | 1.06(0.38-1.80) | 173.97(97.03-281.25) | 0.57(0.48-0.66) | |
| Malta | 6.80(2.72-10.55) | 1.87(0.74-2.92) | 20.72(8.67-33.28) | 1.74(0.73-2.80) | 204.73(140.58-293.16) | -0.04(-0.20-0.11) | |
| Marshall Islands | 0.16(0.07-0.26) | 1.65(0.67-2.81) | 0.41(0.20-0.66) | 2.49(1.19-3.89) | 157.72(87.68-277.94) | 1.18(1.03-1.32) | |
| Mauritania | 9.70(4.26-15.97) | 1.64(0.73-2.73) | 29.61(13.50-47.77) | 2.17(0.97-3.51) | 205.35(105.59-350.81) | 0.72(0.61-0.83) | |
| Mauritius | 7.07(2.70-11.20) | 1.63(0.61-2.60) | 27.50(11.23-43.10) | 1.76(0.72-2.76) | 288.81(229.35-376.03) | 0.05(-0.23-0.32) | |
| Mexico | 422.36(181.61-669.50) | 1.59(0.68-2.53) | 1841.61(889.16-2803.91) | 1.73(0.83-2.64) | 336.03(260.15-461.08) | 0.47(0.37-0.58) | |
| Micronesia (Federated States of) | 0.64(0.27-1.10) | 2.01(0.80-3.66) | 1.08(0.54-1.75) | 2.67(1.25-4.49) | 67.74(19.34-147.02) | 0.85(0.80-0.90) | |
| Monaco | 1.41(0.58-2.26) | 1.63(0.66-2.59) | 2.15(0.97-3.40) | 1.59(0.74-2.51) | 51.79(4.31-131.65) | -0.00(-0.20-0.20) | |
| Mongolia | 5.27(2.03-8.59) | 0.69(0.27-1.16) | 9.74(4.22-15.74) | 0.70(0.30-1.14) | 84.82(32.82-150.99) | -0.39(-0.57--0.21) | |
| Montenegro | 26.25(12.25-40.83) | 4.82(2.24-7.57) | 56.13(28.70-86.27) | 7.15(3.54-11.12) | 113.85(55.70-202.72) | 1.63(1.15-2.11) | |
| Morocco | 113.56(40.57-197.67) | 1.12(0.39-2.00) | 388.42(163.43-628.34) | 1.65(0.69-2.66) | 242.03(151.08-387.94) | 1.31(1.17-1.45) | |
| Mozambique | 27.62(7.68-55.68) | 0.81(0.22-1.71) | 85.13(28.89-156.22) | 1.41(0.46-2.63) | 208.24(119.18-364.55) | 2.21(2.07-2.35) | |
| Myanmar | 157.62(52.78-288.04) | 1.29(0.43-2.36) | 524.96(190.54-864.96) | 1.65(0.59-2.73) | 233.06(130.13-410.07) | 0.55(0.44-0.66) | |
| Namibia | 3.24(1.20-5.31) | 1.01(0.36-1.67) | 11.04(5.11-17.79) | 1.42(0.65-2.34) | 240.54(143.08-409.75) | 1.11(0.89-1.32) | |
| Nauru | 0.07(0.03-0.10) | 2.57(1.09-4.11) | 0.12(0.06-0.22) | 3.73(1.57-7.73) | 85.14(31.41-209.52) | 1.24(1.18-1.31) | |
| Nepal | 27.17(7.12-52.36) | 0.54(0.14-1.06) | 119.26(36.08-224.19) | 0.79(0.23-1.49) | 338.98(187.67-601.56) | 1.35(1.29-1.41) | |
| Netherlands | 474.60(186.57-745.68) | 2.35(0.92-3.70) | 882.04(393.74-1388.94) | 2.12(0.95-3.33) | 85.85(56.49-121.09) | -1.01(-1.35--0.68) | |
| New Zealand | 108.81(47.33-164.44) | 3.07(1.31-4.66) | 252.72(116.60-391.25) | 2.61(1.20-4.04) | 132.26(100.46-175.21) | -0.26(-0.45--0.06) | |
| Nicaragua | 12.93(5.72-20.23) | 1.14(0.49-1.79) | 51.83(24.46-81.50) | 1.38(0.65-2.15) | 300.85(202.96-451.76) | 0.95(0.68-1.22) | |
| Niger | 9.66(2.79-18.74) | 0.82(0.24-1.66) | 37.51(10.53-70.33) | 1.00(0.30-1.83) | 288.34(174.99-436.05) | 0.70(0.64-0.76) | |
| Nigeria | 345.14(134.71-597.98) | 1.37(0.54-2.40) | 830.66(360.22-1305.08) | 1.68(0.73-2.63) | 140.67(70.10-261.03) | 0.63(0.55-0.72) | |
| Niue | 0.06(0.02-0.09) | 2.22(0.91-3.77) | 0.05(0.03-0.08) | 2.85(1.34-4.43) | -2.94(-30.09-35.29) | 0.61(0.50-0.72) | |
| North Macedonia | 26.39(11.67-40.58) | 1.81(0.80-2.80) | 60.67(28.59-101.82) | 2.97(1.32-4.78) | 129.88(62.23-225.54) | 1.38(0.78-1.97) | |
| Northern Mariana Islands | 0.14(0.07-0.22) | 1.74(0.77-3.00) | 0.85(0.45-1.27) | 2.89(1.48-4.38) | 521.08(355.39-786.73) | 1.52(1.18-1.87) | |
| Norway | 188.00(77.59-288.78) | 2.40(0.99-3.68) | 266.98(112.57-424.65) | 2.06(0.87-3.26) | 42.01(21.53-64.08) | -0.65(-0.90--0.40) | |
| Oman | 3.94(1.49-7.11) | 0.93(0.35-1.70) | 18.24(9.60-27.93) | 1.84(0.94-2.90) | 362.63(180.98-686.74) | 3.15(2.68-3.63) | |
| Pakistan | 309.52(95.27-592.59) | 0.82(0.25-1.57) | 1001.37(390.09-1763.53) | 1.42(0.54-2.53) | 223.53(130.55-353.26) | 1.62(1.35-1.89) | |
| Palau | 0.08(0.03-0.12) | 1.25(0.55-2.10) | 0.20(0.10-0.31) | 1.76(0.87-2.68) | 170.04(92.45-264.72) | 1.21(1.09-1.32) | |
| Palestine | 8.99(3.88-14.89) | 1.58(0.68-2.67) | 24.50(12.64-37.12) | 1.88(0.95-2.86) | 172.47(93.49-314.73) | 0.32(0.13-0.52) | |
| Panama | 18.73(8.62-28.53) | 1.50(0.69-2.30) | 89.26(42.60-136.03) | 1.87(0.89-2.85) | 376.52(272.59-519.60) | 0.81(0.71-0.91) | |
| Papua New Guinea | 5.67(1.59-10.92) | 0.61(0.16-1.19) | 22.49(7.62-41.99) | 0.74(0.22-1.45) | 296.89(178.81-484.25) | 0.58(0.55-0.62) | |
| Paraguay | 27.98(10.86-46.22) | 1.52(0.59-2.52) | 104.94(45.09-169.43) | 2.07(0.88-3.36) | 275.08(175.12-450.59) | 1.34(1.18-1.50) | |
| Peru | 78.78(29.56-139.99) | 0.78(0.29-1.39) | 402.72(171.42-668.00) | 1.20(0.51-1.97) | 411.19(246.76-721.59) | 1.69(1.42-1.96) | |
| Philippines | 193.31(72.06-330.12) | 1.40(0.51-2.34) | 687.25(250.25-1173.71) | 1.34(0.48-2.33) | 255.52(171.56-370.01) | 0.16(0.03-0.30) | |
| Poland | 966.20(443.51-1443.95) | 2.59(1.17-3.86) | 1389.32(693.55-2116.74) | 1.72(0.86-2.62) | 43.79(17.89-76.98) | -0.94(-1.59--0.29) | |
| Portugal | 169.67(68.80-263.22) | 1.54(0.61-2.42) | 378.90(168.66-589.46) | 1.12(0.51-1.74) | 123.31(80.60-194.53) | -1.23(-1.43--1.02) | |
| Puerto Rico | 56.76(25.92-87.25) | 1.96(0.90-3.01) | 141.55(69.29-217.34) | 1.41(0.69-2.15) | 149.38(93.47-219.53) | -1.16(-1.27--1.04) | |
| Qatar | 0.84(0.27-1.45) | 2.60(0.78-4.54) | 4.35(1.90-7.76) | 1.74(0.51-3.13) | 419.66(223.88-737.44) | -2.04(-2.75--1.33) | |
| Republic of Korea | 164.81(56.14-293.61) | 0.99(0.34-1.82) | 791.54(271.02-1402.67) | 0.87(0.30-1.54) | 380.27(128.39-644.68) | -0.59(-0.72--0.45) | |
| Republic of Moldova | 41.53(19.25-62.66) | 1.49(0.69-2.21) | 95.02(48.58-141.80) | 1.55(0.79-2.31) | 128.82(91.08-182.35) | -0.04(-0.23-0.14) | |
| Romania | 285.16(127.65-418.55) | 1.48(0.65-2.16) | 605.88(285.06-884.46) | 1.42(0.67-2.08) | 112.47(80.05-159.95) | -0.55(-0.73--0.36) | |
| Russian Federation | 1966.26(898.90-3059.55) | 1.49(0.68-2.33) | 4418.66(2219.98-6375.44) | 1.81(0.91-2.61) | 124.72(87.96-173.89) | 0.63(0.49-0.77) | |
| Rwanda | 13.82(3.64-27.20) | 0.90(0.24-1.84) | 30.42(7.00-62.39) | 0.91(0.20-1.88) | 120.09(37.95-229.47) | -0.54(-0.80--0.28) | |
| Saint Kitts and Nevis | 0.77(0.32-1.23) | 2.58(1.07-4.16) | 1.09(0.51-1.69) | 2.59(1.14-4.00) | 41.28(7.66-91.35) | 0.34(0.12-0.55) | |
| Saint Lucia | 1.95(0.72-3.04) | 3.35(1.25-5.27) | 5.12(2.01-8.44) | 2.32(0.92-3.82) | 163.01(100.89-240.83) | -1.83(-2.20--1.47) | |
| Saint Vincent and the Grenadines | 1.21(0.44-2.06) | 2.13(0.77-3.65) | 2.47(1.01-3.92) | 2.17(0.88-3.48) | 103.69(63.67-168.91) | 0.20(-0.05-0.46) | |
| Samoa | 1.06(0.46-1.75) | 1.81(0.73-3.09) | 2.48(1.23-3.94) | 2.37(1.13-3.83) | 133.35(76.89-222.83) | 0.80(0.74-0.87) | |
| San Marino | 0.83(0.35-1.34) | 2.19(0.91-3.52) | 1.35(0.54-2.32) | 1.12(0.45-1.91) | 62.27(4.61-165.47) | -1.36(-1.75--0.96) | |
| Sao Tome and Principe | 0.51(0.17-0.96) | 1.11(0.35-2.18) | 1.18(0.51-1.87) | 1.86(0.81-3.00) | 132.92(50.51-278.84) | 1.98(1.83-2.12) | |
| Saudi Arabia | 33.68(14.40-56.65) | 0.97(0.41-1.63) | 100.25(54.12-152.37) | 1.36(0.75-2.06) | 197.67(97.62-367.54) | 1.00(0.90-1.10) | |
| Senegal | 25.61(9.03-44.00) | 1.36(0.47-2.33) | 88.93(34.38-144.08) | 1.91(0.74-3.10) | 247.28(154.58-425.64) | 0.97(0.92-1.03) | |
| Serbia | 172.26(71.89-264.10) | 2.45(1.04-3.78) | 340.47(164.44-501.52) | 1.94(0.94-2.86) | 97.65(56.35-170.10) | -1.27(-1.62--0.91) | |
| Seychelles | 0.86(0.32-1.42) | 1.59(0.59-2.65) | 1.70(0.77-2.73) | 1.99(0.89-3.23) | 97.94(50.25-179.19) | 0.97(0.78-1.16) | |
| Sierra Leone | 17.90(5.80-31.48) | 1.36(0.45-2.40) | 31.99(12.11-51.57) | 1.48(0.56-2.35) | 78.73(36.18-149.49) | 0.21(0.18-0.24) | |
| Singapore | 9.83(3.44-15.95) | 0.60(0.21-0.98) | 25.04(10.37-41.59) | 0.31(0.13-0.52) | 154.84(94.55-235.51) | -2.70(-2.99--2.40) | |
| Slovakia | 132.80(63.00-197.77) | 2.49(1.18-3.72) | 232.90(116.58-348.32) | 2.47(1.23-3.70) | 75.38(31.82-129.25) | 0.23(0.11-0.34) | |
| Slovenia | 35.72(16.30-52.64) | 1.52(0.69-2.25) | 86.53(41.98-130.59) | 1.50(0.73-2.26) | 142.22(100.90-187.65) | 0.29(-0.14-0.73) | |
| Solomon Islands | 0.53(0.15-0.99) | 0.80(0.22-1.63) | 2.42(0.86-4.64) | 1.33(0.43-2.81) | 352.72(222.84-561.58) | 1.58(1.47-1.69) | |
| Somalia | 7.22(1.66-14.74) | 0.63(0.14-1.31) | 18.05(4.45-39.69) | 0.67(0.16-1.49) | 150.06(70.65-280.69) | 0.45(0.36-0.53) | |
| South Africa | 152.70(66.70-244.95) | 1.02(0.44-1.65) | 504.26(245.40-735.38) | 1.66(0.81-2.47) | 230.24(152.47-342.67) | 1.52(1.14-1.89) | |
| South Sudan | 12.47(2.69-24.61) | 0.75(0.17-1.49) | 18.57(5.01-36.59) | 0.88(0.24-1.74) | 48.84(6.23-111.18) | 0.53(0.50-0.57) | |
| Spain | 1031.20(444.37-1565.25) | 2.03(0.88-3.09) | 2363.75(1093.08-3764.63) | 1.60(0.75-2.56) | 129.22(90.96-182.77) | -0.66(-0.75--0.57) | |
| Sri Lanka | 64.24(22.82-105.64) | 1.11(0.40-1.86) | 266.89(105.93-449.88) | 1.37(0.54-2.29) | 315.44(166.74-528.23) | 1.40(1.09-1.71) | |
| Sudan | 55.70(20.19-106.10) | 1.00(0.35-1.88) | 163.64(77.32-264.67) | 1.32(0.61-2.11) | 193.81(101.00-376.70) | 0.90(0.77-1.02) | |
| Suriname | 3.20(1.13-5.44) | 1.49(0.52-2.55) | 7.95(3.14-13.66) | 1.45(0.56-2.52) | 148.60(69.88-275.43) | 0.22(0.07-0.38) | |
| Sweden | 313.96(126.75-487.23) | 1.82(0.74-2.81) | 1019.10(454.77-1640.84) | 3.41(1.51-5.52) | 224.60(163.49-300.43) | 2.40(2.09-2.70) | |
| Switzerland | 105.52(43.15-166.01) | 0.91(0.37-1.42) | 248.90(111.76-409.99) | 0.97(0.44-1.57) | 135.87(82.89-203.25) | 0.64(0.48-0.81) | |
| Syrian Arab Republic | 51.05(21.43-84.55) | 1.42(0.59-2.38) | 131.16(62.38-215.35) | 1.82(0.85-3.00) | 156.94(64.12-322.58) | 0.45(0.25-0.64) | |
| Taiwan (Province of China) | 125.02(43.44-202.43) | 1.58(0.55-2.57) | 510.70(215.41-830.49) | 1.08(0.46-1.76) | 308.48(227.85-435.66) | -1.48(-1.86--1.10) | |
| Tajikistan | 9.02(3.89-16.56) | 0.41(0.17-0.76) | 14.86(6.55-23.32) | 0.43(0.19-0.68) | 64.76(6.70-138.13) | -0.02(-0.40-0.36) | |
| Thailand | 272.83(94.26-482.04) | 1.29(0.44-2.30) | 1458.10(571.81-2510.04) | 1.32(0.52-2.26) | 434.44(254.10-734.79) | -0.32(-0.56--0.07) | |
| Timor-Leste | 1.38(0.43-2.56) | 1.00(0.31-1.86) | 6.97(2.56-11.71) | 1.40(0.52-2.40) | 405.49(236.44-686.21) | 1.26(1.10-1.41) | |
| Togo | 8.29(2.88-14.27) | 1.34(0.45-2.30) | 29.77(11.91-48.16) | 1.82(0.73-2.89) | 259.00(159.71-426.82) | 0.95(0.90-1.00) | |
| Tokelau | 0.02(0.01-0.03) | 1.70(0.71-3.02) | 0.04(0.02-0.06) | 2.55(1.12-4.36) | 98.32(32.42-224.96) | 1.27(1.13-1.41) | |
| Tonga | 0.45(0.19-0.80) | 1.14(0.45-2.09) | 1.39(0.68-2.32) | 1.92(0.94-3.22) | 205.30(116.50-354.24) | 1.73(1.50-1.95) | |
| Trinidad and Tobago | 12.08(4.99-18.94) | 2.27(0.93-3.64) | 34.36(15.97-56.59) | 1.95(0.91-3.25) | 184.50(111.43-287.77) | -0.38(-0.50--0.26) | |
| Tunisia | 27.65(9.81-46.03) | 1.01(0.35-1.73) | 151.81(64.41-257.87) | 1.49(0.64-2.59) | 449.02(250.00-805.43) | 1.12(1.05-1.19) | |
| Turkmenistan | 9.62(4.18-14.87) | 0.72(0.30-1.15) | 33.23(14.52-54.13) | 1.12(0.48-1.83) | 245.53(161.13-371.04) | 1.17(0.98-1.37) | |
| Tuvalu | 0.06(0.03-0.10) | 1.54(0.61-2.81) | 0.17(0.08-0.28) | 2.46(1.09-4.05) | 184.81(110.53-302.05) | 1.39(1.21-1.56) | |
| Türkiye | 289.84(125.47-454.43) | 1.24(0.53-1.97) | 1093.05(563.02-1671.39) | 1.44(0.74-2.22) | 277.12(166.76-441.14) | 0.84(0.38-1.30) | |
| Uganda | 33.69(8.49-67.49) | 0.87(0.22-1.80) | 79.98(21.53-159.77) | 0.92(0.24-1.87) | 137.40(64.20-261.63) | -0.06(-0.15-0.04) | |
| Ukraine | 852.39(396.92-1281.26) | 1.49(0.70-2.27) | 1333.23(627.27-1986.04) | 1.69(0.81-2.50) | 56.41(18.00-108.13) | -0.00(-0.24-0.24) | |
| United Arab Emirates | 2.57(1.03-4.42) | 1.34(0.53-2.34) | 12.72(6.61-19.48) | 1.55(0.72-2.47) | 395.47(227.54-740.39) | 3.27(2.24-4.30) | |
| United Kingdom | 2142.21(943.57-3190.47) | 2.29(1.01-3.42) | 3131.74(1500.98-4741.17) | 1.90(0.92-2.88) | 46.19(29.39-71.10) | -0.75(-1.00--0.50) | |
| United Republic of Tanzania | 59.38(17.42-113.30) | 0.97(0.28-1.87) | 180.87(59.33-333.70) | 1.09(0.35-2.01) | 204.61(122.12-356.34) | 0.33(0.10-0.57) | |
| United States of America | 4957.01(2218.96-7545.26) | 1.47(0.66-2.24) | 12991.65(6549.67-19666.05) | 1.93(0.98-2.92) | 162.09(125.63-225.82) | 0.65(0.51-0.80) | |
| United States Virgin Islands | 1.19(0.53-1.86) | 2.54(1.12-3.99) | 2.74(1.33-4.42) | 1.78(0.88-2.93) | 128.98(63.93-220.49) | -0.84(-1.06--0.63) | |
| Uruguay | 42.06(18.47-64.51) | 1.17(0.51-1.80) | 103.96(48.31-157.82) | 1.41(0.66-2.15) | 147.16(102.82-210.16) | 0.76(0.64-0.89) | |
| Uzbekistan | 28.92(9.00-62.90) | 0.30(0.09-0.66) | 85.38(41.10-128.51) | 0.49(0.23-0.73) | 195.19(64.26-434.02) | 1.81(1.59-2.04) | |
| Vanuatu | 0.51(0.17-0.86) | 1.60(0.53-2.80) | 1.80(0.75-2.92) | 1.92(0.77-3.12) | 256.53(180.67-379.46) | 0.52(0.43-0.60) | |
| Venezuela (Bolivarian Republic of) | 123.00(52.25-183.75) | 1.73(0.73-2.60) | 480.82(220.37-761.76) | 1.84(0.84-2.91) | 290.93(203.18-404.23) | 0.08(-0.08-0.24) | |
| Viet Nam | 368.81(128.84-653.84) | 1.26(0.45-2.25) | 1418.64(483.77-2377.60) | 2.06(0.71-3.48) | 284.65(178.89-434.41) | 1.72(1.60-1.83) | |
| Yemen | 22.07(6.96-43.43) | 0.89(0.27-1.77) | 94.90(40.93-163.90) | 1.20(0.52-2.11) | 329.89(182.41-637.22) | 0.91(0.80-1.01) | |
| Zambia | 14.54(4.49-25.75) | 0.92(0.28-1.64) | 61.51(20.49-126.75) | 1.54(0.52-3.06) | 323.13(134.27-628.05) | 1.90(1.54-2.25) | |
| Zimbabwe | 21.17(7.83-35.07) | 1.02(0.37-1.71) | 46.98(19.11-75.83) | 1.34(0.50-2.18) | 121.94(39.93-219.99) | 1.40(0.99-1.80) | |

^AF/AFL, atrial fibrillation/flutter; EAPC, estimated annual percentage changes; GBD, Global Burden of Disease, Injuries, and Risk Factors Study; ASDR, age-standardized death rate; UI, uncertainty interval; PC, percentage change.^

**Supplemental Table S3** Numbers and age standardized disability-adjusted life years rates of atrial fibrillation/flutter related to metabolic risks in 1990 and 2021, and their percentage change of disability-adjusted life years numbers, estimated annual percentage changes in age standardized rate from 1990 to 2021, by Global Burden of Disease region and 204 countries

|  | 1990 | | 2021 | |  | |  |
| --- | --- | --- | --- | --- | --- | --- | --- |
|  | **Number of DALYs (95% UI)** | **ASR of DALYs per 100000 people (95% UI)** | **Number of DALYs (95% UI)** | **ASR of DALYs per 100000 people (95% UI)** | **PC number of DALYs 1990-2021 (95% UI)** | **EAPC of ASR of DALYs 1990-2021 (95% CI)** | |
| **Region** |  |  |  |  |  |  | |
| Andean Latin America | 3855.26(1522.57-6522.76) | 21.41(8.45-36.55) | 18160.93(8133.07-29004.24) | 32.21(14.38-51.31) | 371.07(282.50-503.63) | 1.61(1.46-1.76) | |
| Australasia | 12968.48(5503.77-20039.48) | 57.27(24.26-88.45) | 31106.06(14703.80-47988.89) | 52.62(24.91-81.13) | 139.86(106.84-194.24) | -0.27(-0.32--0.21) | |
| Caribbean | 8321.23(3282.55-13501.68) | 35.77(14.10-57.76) | 20848.75(9456.43-32782.02) | 38.15(17.22-60.08) | 150.55(124.65-199.34) | 0.26(0.22-0.30) | |
| Central Asia | 10226.45(4404.26-16262.44) | 24.21(10.43-38.58) | 20801.38(9538.67-32682.81) | 29.75(13.61-45.78) | 103.41(87.25-125.32) | 0.65(0.55-0.75) | |
| Central Europe | 64563.35(29652.99-96544.82) | 46.94(21.65-70.22) | 105572.27(50376.07-157343.72) | 44.45(21.17-66.52) | 63.52(50.93-84.30) | -0.20(-0.38--0.03) | |
| Central Latin America | 25122.45(10767.90-39603.29) | 36.26(15.47-57.11) | 101612.81(47341.48-155377.02) | 42.81(19.81-65.43) | 304.47(265.21-377.32) | 0.59(0.56-0.63) | |
| Central Sub-Saharan Africa | 4702.64(1648.10-8039.07) | 30.18(10.57-51.38) | 12484.69(4561.45-20808.51) | 32.53(12.00-54.60) | 165.48(115.99-238.49) | 0.12(0.03-0.20) | |
| East Asia | 132866.27(44632.77-230699.09) | 24.15(8.21-41.95) | 551927.47(223379.50-907787.91) | 28.56(11.45-47.34) | 315.40(227.93-468.44) | 0.48(0.37-0.59) | |
| Eastern Europe | 95507.71(42629.67-146477.83) | 37.19(16.41-56.71) | 160426.34(78219.75-241140.48) | 44.36(21.64-66.53) | 67.97(51.62-94.44) | 0.54(0.45-0.62) | |
| Eastern Sub-Saharan Africa | 10238.77(3309.78-18248.25) | 18.34(5.96-32.54) | 31276.93(11190.14-52397.24) | 23.71(8.40-40.12) | 205.48(165.67-273.35) | 0.85(0.79-0.91) | |
| High-income Asia Pacific | 53722.81(18805.15-87127.98) | 28.48(9.96-46.06) | 106326.56(39409.04-177937.09) | 19.86(7.40-33.15) | 97.92(75.45-127.06) | -1.66(-1.98--1.35) | |
| High-income North America | 164797.95(73769.68-257074.90) | 44.84(20.08-69.52) | 363764.69(188691.85-558805.31) | 51.45(26.67-79.09) | 120.73(85.47-182.40) | 0.24(0.13-0.35) | |
| North Africa and Middle East | 29666.87(13245.91-46819.84) | 23.84(10.58-37.35) | 102427.80(53351.71-155201.39) | 29.03(15.30-43.90) | 245.26(183.17-338.49) | 0.62(0.52-0.71) | |
| Oceania | 528.39(221.86-895.79) | 24.24(9.72-41.37) | 1826.67(849.74-2906.08) | 29.97(13.43-48.01) | 245.71(202.13-301.72) | 0.68(0.62-0.73) | |
| South Asia | 85543.24(29551.87-149913.80) | 19.92(6.87-35.16) | 326968.47(119794.87-541253.40) | 26.86(9.99-44.94) | 282.23(227.58-350.78) | 1.05(0.97-1.12) | |
| Southeast Asia | 61042.29(21376.62-101000.44) | 31.65(11.02-51.61) | 209694.48(81561.60-341315.16) | 39.56(15.39-64.06) | 243.52(200.19-297.02) | 0.76(0.66-0.85) | |
| Southern Latin America | 11011.48(4827.00-17462.60) | 26.44(11.63-41.66) | 26752.10(12753.97-40292.84) | 29.35(14.02-44.15) | 142.95(113.36-189.27) | 0.91(0.61-1.21) | |
| Southern Sub-Saharan Africa | 6320.01(2676.09-10059.34) | 27.89(11.64-44.05) | 17442.06(8668.98-26099.99) | 37.60(18.51-55.91) | 175.98(141.08-236.31) | 0.99(0.78-1.19) | |
| Tropical Latin America | 29001.17(11749.59-46410.04) | 39.05(15.85-63.20) | 109392.22(49970.77-171703.07) | 44.15(20.24-68.97) | 277.20(245.40-348.51) | 0.36(0.31-0.41) | |
| Western Europe | 306976.55(130648.68-479080.18) | 50.95(21.65-79.21) | 519013.27(243576.21-805623.61) | 47.14(22.27-73.95) | 69.07(53.48-92.08) | -0.31(-0.36--0.26) | |
| Western Sub-Saharan Africa | 15296.24(5774.84-25343.85) | 24.20(9.09-40.64) | 43974.50(18238.81-69228.19) | 31.94(13.29-50.07) | 187.49(140.50-256.47) | 0.96(0.90-1.03) | |
| **Countries** |  |  |  |  |  |  | |
| Afghanistan | 1009.95(352.41-1818.07) | 18.39(6.33-33.47) | 1611.73(685.62-2736.54) | 22.89(9.63-38.12) | 59.59(24.39-113.17) | 0.75(0.68-0.83) | |
| Albania | 655.77(276.43-1016.04) | 37.78(15.95-58.75) | 1796.36(798.32-2715.59) | 41.79(18.32-63.39) | 173.93(124.67-242.06) | 0.53(0.43-0.63) | |
| Algeria | 2328.25(942.08-3784.24) | 31.22(12.12-51.05) | 8896.01(4419.66-14094.80) | 35.98(17.59-56.57) | 282.09(195.42-421.24) | 0.78(0.67-0.90) | |
| American Samoa | 7.88(4.13-12.16) | 42.08(21.71-67.06) | 24.56(13.11-36.89) | 59.11(30.60-90.45) | 211.74(161.07-277.54) | 1.12(1.02-1.22) | |
| Andorra | 23.51(9.17-39.08) | 45.79(18.25-76.02) | 60.59(25.38-99.65) | 36.73(15.27-60.36) | 157.67(107.86-219.43) | -0.68(-0.77--0.58) | |
| Angola | 760.27(264.80-1297.63) | 28.24(9.89-48.48) | 2791.53(1021.62-4701.62) | 34.40(12.74-58.01) | 267.17(178.73-397.59) | 0.54(0.47-0.60) | |
| Antigua and Barbuda | 24.31(10.05-38.46) | 42.03(17.51-66.21) | 41.98(19.84-66.30) | 45.06(20.66-70.82) | 72.68(41.60-119.14) | 0.27(0.14-0.40) | |
| Argentina | 7047.08(3052.65-11181.10) | 24.48(10.74-38.84) | 15361.89(7323.99-23163.20) | 26.37(12.61-39.78) | 117.99(81.33-178.38) | 0.90(0.57-1.24) | |
| Armenia | 521.46(232.36-833.27) | 21.55(9.54-33.82) | 1318.24(600.42-2060.12) | 30.03(13.73-47.26) | 152.80(118.77-200.54) | 1.32(1.14-1.51) | |
| Australia | 10588.76(4480.21-16524.64) | 56.27(23.70-87.70) | 26206.96(12296.43-40168.87) | 52.44(24.55-80.19) | 147.50(109.73-209.23) | -0.26(-0.33--0.19) | |
| Austria | 5416.17(2283.96-8449.45) | 43.71(18.52-67.93) | 13468.62(6068.72-21220.67) | 63.90(28.83-100.04) | 148.67(111.93-195.85) | 1.41(1.20-1.62) | |
| Azerbaijan | 935.19(389.37-1542.07) | 21.35(8.87-34.86) | 2324.35(1035.50-3763.79) | 26.97(11.90-43.43) | 148.54(107.91-211.44) | 1.00(0.89-1.10) | |
| Bahamas | 56.62(24.75-88.50) | 41.90(18.01-65.94) | 170.40(81.63-259.59) | 47.74(22.76-72.93) | 200.97(146.00-290.50) | 0.46(0.35-0.58) | |
| Bahrain | 35.39(13.88-57.99) | 38.94(13.88-65.72) | 180.57(89.43-285.00) | 38.14(16.11-62.65) | 410.18(288.78-616.92) | -0.24(-0.51-0.02) | |
| Bangladesh | 7467.06(2288.12-13552.82) | 19.62(6.05-35.63) | 33703.06(11238.53-57763.74) | 29.09(9.77-49.97) | 351.36(257.90-498.27) | 1.27(1.04-1.50) | |
| Barbados | 113.27(44.64-183.29) | 36.60(14.62-59.08) | 247.40(115.25-385.18) | 47.27(22.16-73.38) | 118.42(86.78-181.02) | 0.91(0.74-1.08) | |
| Belarus | 4375.78(1902.82-6987.23) | 34.97(15.15-55.55) | 7076.65(3396.95-10970.43) | 42.92(20.70-66.62) | 61.72(35.62-97.43) | 0.56(0.49-0.63) | |
| Belgium | 5836.71(2275.39-9552.42) | 36.55(14.11-59.97) | 10556.01(4678.91-16491.70) | 38.95(17.34-60.62) | 80.86(55.96-120.69) | 0.37(0.21-0.53) | |
| Belize | 30.18(13.27-48.70) | 33.36(14.61-53.82) | 108.41(53.40-167.33) | 41.47(20.09-64.13) | 259.16(196.23-364.36) | 0.60(0.32-0.88) | |
| Benin | 333.66(125.39-567.28) | 20.25(7.51-34.38) | 996.45(404.78-1586.17) | 25.85(10.30-41.13) | 198.64(141.53-277.47) | 0.86(0.81-0.90) | |
| Bermuda | 24.11(10.22-39.68) | 42.95(17.74-70.37) | 58.19(27.41-90.08) | 38.55(18.09-59.57) | 141.41(101.41-213.96) | -0.43(-0.49--0.36) | |
| Bhutan | 34.51(12.15-62.64) | 19.78(6.66-35.58) | 150.92(57.15-255.45) | 27.87(10.54-47.26) | 337.30(241.31-468.38) | 1.23(1.18-1.28) | |
| Bolivia (Plurinational State of) | 662.61(250.05-1182.60) | 25.81(9.64-46.07) | 2732.79(1157.03-4601.05) | 36.14(15.15-60.10) | 312.43(215.97-474.22) | 1.33(1.22-1.44) | |
| Bosnia and Herzegovina | 1210.58(522.93-1880.21) | 35.03(14.77-54.44) | 2593.00(1250.21-4001.88) | 40.19(19.41-62.08) | 114.20(77.03-165.63) | 0.55(0.48-0.62) | |
| Botswana | 104.03(41.41-171.91) | 25.38(9.88-41.97) | 339.12(151.01-538.55) | 30.39(13.56-47.88) | 225.99(157.55-371.89) | 0.74(0.59-0.89) | |
| Brazil | 28216.55(11435.61-45150.39) | 39.05(15.85-63.21) | 106829.15(48903.79-167617.73) | 44.07(20.25-68.93) | 278.60(246.84-350.82) | 0.35(0.30-0.40) | |
| Brunei Darussalam | 39.46(13.85-65.03) | 46.84(15.95-77.74) | 105.82(47.32-169.30) | 37.88(15.14-62.95) | 168.16(111.69-263.51) | -0.41(-0.59--0.23) | |
| Bulgaria | 5035.36(2311.13-7718.54) | 48.42(22.74-73.15) | 8135.86(3923.12-12472.50) | 53.79(26.19-82.45) | 61.57(35.98-91.88) | 0.36(0.26-0.46) | |
| Burkina Faso | 666.35(216.29-1170.94) | 21.74(7.08-37.99) | 1956.36(707.64-3353.76) | 29.66(10.89-49.89) | 193.59(132.39-270.63) | 1.26(1.15-1.38) | |
| Burundi | 344.44(103.57-669.79) | 17.88(5.32-34.23) | 782.93(250.59-1459.25) | 21.71(6.83-41.25) | 127.31(74.23-195.79) | 0.48(0.43-0.54) | |
| Cabo Verde | 55.90(19.43-94.93) | 23.35(8.28-39.52) | 146.76(62.54-240.20) | 35.59(15.07-58.49) | 162.52(81.71-273.53) | 1.31(1.17-1.46) | |
| Cambodia | 747.41(259.91-1307.75) | 23.20(8.19-40.31) | 2294.08(764.04-4054.35) | 26.62(9.08-45.96) | 206.94(132.15-296.30) | 0.41(0.30-0.52) | |
| Cameroon | 734.76(286.60-1239.81) | 23.62(8.85-40.11) | 3375.28(1583.19-5217.01) | 39.14(17.82-59.83) | 359.37(263.48-520.92) | 1.64(1.34-1.94) | |
| Canada | 17801.93(8004.83-28663.35) | 54.56(24.45-88.02) | 33560.16(15705.11-51994.96) | 42.07(19.54-65.60) | 88.52(62.54-131.92) | -0.94(-1.09--0.78) | |
| Central African Republic | 229.47(76.32-409.24) | 29.47(9.47-52.03) | 446.31(160.56-791.78) | 30.56(10.81-54.45) | 94.49(54.07-151.35) | 0.07(0.05-0.10) | |
| Chad | 431.47(148.71-758.22) | 18.74(6.48-33.49) | 957.96(361.10-1633.50) | 23.57(8.64-39.99) | 122.02(76.03-181.86) | 0.75(0.72-0.78) | |
| Chile | 2926.07(1308.53-4486.16) | 32.81(14.69-50.38) | 9446.09(4293.69-14153.62) | 35.83(16.27-53.71) | 222.82(190.32-276.86) | 0.83(0.48-1.17) | |
| China | 125423.49(42139.39-219272.08) | 23.86(8.16-41.81) | 531011.93(214701.35-876899.81) | 28.62(11.41-47.59) | 323.38(229.91-484.11) | 0.54(0.42-0.65) | |
| Colombia | 5076.74(1995.88-8089.30) | 35.09(13.85-55.57) | 22655.73(10239.01-34816.54) | 40.64(18.32-62.47) | 346.27(285.32-449.48) | 0.44(0.37-0.50) | |
| Comoros | 32.29(10.00-58.54) | 21.31(6.59-39.45) | 103.17(38.56-178.71) | 25.93(9.61-45.94) | 219.51(149.03-323.25) | 0.53(0.49-0.57) | |
| Congo | 306.01(111.47-519.45) | 39.71(14.23-67.73) | 807.59(337.21-1267.96) | 42.59(17.00-67.62) | 163.91(105.08-246.71) | 0.09(0.01-0.17) | |
| Cook Islands | 4.38(2.20-6.91) | 40.66(19.63-64.42) | 13.35(6.65-20.85) | 52.93(26.27-83.17) | 204.52(146.92-292.73) | 0.84(0.78-0.91) | |
| Costa Rica | 662.35(278.96-1048.20) | 40.94(17.15-64.59) | 2393.31(1121.55-3708.28) | 43.06(20.09-66.69) | 261.34(216.48-336.14) | 0.03(-0.07-0.13) | |
| Croatia | 1562.02(722.86-2392.74) | 28.83(13.18-43.97) | 3122.71(1561.82-4738.85) | 31.65(15.94-48.09) | 99.91(72.90-138.84) | 0.66(0.35-0.97) | |
| Cuba | 3179.32(1164.37-5308.46) | 33.11(12.07-54.89) | 7301.27(3445.41-11861.20) | 34.87(16.38-56.85) | 129.65(89.46-195.68) | 0.17(0.10-0.24) | |
| Cyprus | 435.92(173.05-703.69) | 75.35(29.20-122.86) | 844.94(372.91-1329.33) | 46.35(20.78-73.45) | 93.83(43.73-171.63) | -1.63(-1.86--1.41) | |
| Czechia | 5724.26(2707.70-8827.52) | 42.16(19.92-65.33) | 12697.97(6191.64-19417.48) | 54.17(26.54-82.97) | 121.83(86.61-169.30) | 1.09(0.88-1.30) | |
| Côte d'Ivoire | 636.06(241.07-1037.74) | 26.03(9.40-42.43) | 2430.90(993.46-3844.20) | 32.72(12.91-51.95) | 282.18(208.20-394.99) | 0.72(0.64-0.81) | |
| Democratic People's Republic of Korea | 3449.93(1108.16-5934.31) | 29.23(9.23-50.12) | 8821.60(3203.29-15138.37) | 31.60(11.41-53.85) | 155.70(105.13-244.62) | 0.36(0.29-0.43) | |
| Democratic Republic of the Congo | 3168.64(1058.35-5556.30) | 29.33(9.65-51.18) | 7904.96(2881.41-13467.43) | 30.62(10.99-53.19) | 149.47(95.74-228.91) | 0.01(-0.08-0.10) | |
| Denmark | 4235.11(1706.26-6636.24) | 48.70(19.77-75.95) | 7017.99(2922.87-11216.42) | 53.61(22.77-85.98) | 65.71(47.06-91.36) | 0.06(-0.25-0.37) | |
| Djibouti | 19.13(6.30-34.50) | 21.13(7.01-38.32) | 117.81(41.34-206.59) | 27.33(9.39-48.08) | 515.97(371.22-733.46) | 0.83(0.79-0.88) | |
| Dominica | 28.06(13.20-43.93) | 49.28(23.14-76.58) | 43.80(22.39-67.28) | 56.78(28.98-87.49) | 56.09(25.37-101.78) | 0.47(0.42-0.52) | |
| Dominican Republic | 1011.27(373.79-1696.83) | 34.55(12.48-58.23) | 3532.90(1537.29-5730.93) | 36.65(15.93-59.66) | 249.35(180.36-361.57) | 0.50(0.33-0.68) | |
| Ecuador | 1062.63(438.17-1802.49) | 23.52(9.54-39.43) | 4717.48(2296.12-7394.59) | 30.93(15.15-48.16) | 343.94(257.78-509.72) | 1.16(1.03-1.29) | |
| Egypt | 3814.97(1728.75-6029.17) | 22.80(10.23-36.41) | 12863.34(6923.23-19120.40) | 30.93(16.01-45.17) | 237.18(169.95-343.17) | 1.19(1.12-1.26) | |
| El Salvador | 1019.66(441.20-1629.63) | 36.43(15.77-58.17) | 3003.99(1455.64-4781.28) | 44.95(21.56-71.11) | 194.61(139.26-278.83) | 0.69(0.62-0.77) | |
| Equatorial Guinea | 44.44(15.99-76.45) | 30.79(10.96-53.08) | 155.09(68.24-245.07) | 42.33(17.95-67.16) | 249.01(144.79-430.75) | 1.07(1.00-1.13) | |
| Eritrea | 138.51(43.50-248.51) | 19.01(6.25-34.88) | 456.64(153.09-810.62) | 25.61(8.53-47.58) | 229.69(146.54-355.52) | 1.02(0.98-1.05) | |
| Estonia | 762.20(341.33-1158.02) | 38.23(17.13-57.94) | 1482.73(705.46-2196.87) | 47.80(23.11-70.68) | 94.53(68.85-131.52) | 0.71(0.62-0.79) | |
| Eswatini | 63.60(28.59-102.51) | 30.45(13.82-48.39) | 160.90(81.25-243.32) | 38.94(20.12-57.57) | 152.97(96.91-241.47) | 1.10(0.85-1.35) | |
| Ethiopia | 1410.99(403.55-2822.90) | 9.70(2.86-19.79) | 6121.70(2052.78-10940.84) | 16.74(5.62-30.43) | 333.86(227.92-492.50) | 2.03(1.90-2.16) | |
| Fiji | 101.94(47.31-161.52) | 35.33(16.54-55.45) | 362.69(185.59-543.09) | 60.05(30.24-90.03) | 255.77(184.75-356.35) | 1.66(1.47-1.84) | |
| Finland | 5917.60(2472.77-9193.60) | 80.96(34.01-125.30) | 6989.04(3273.90-11078.42) | 48.57(22.65-76.50) | 18.11(0.30-41.03) | -1.83(-1.92--1.75) | |
| France | 40147.89(16014.11-64444.08) | 45.13(18.06-72.47) | 66142.39(30305.29-102468.66) | 38.75(18.09-59.42) | 64.75(41.08-102.48) | -0.59(-0.68--0.50) | |
| Gabon | 193.80(74.00-325.06) | 41.09(15.43-69.29) | 379.21(172.81-590.39) | 49.07(22.11-76.23) | 95.67(47.56-162.82) | 0.44(0.34-0.54) | |
| Gambia | 62.75(23.13-105.74) | 25.67(9.54-43.07) | 251.35(104.23-401.81) | 34.22(13.78-55.40) | 300.55(210.02-427.99) | 0.90(0.84-0.97) | |
| Georgia | 2124.08(896.82-3364.63) | 35.86(15.14-56.53) | 3137.05(1409.48-4804.55) | 49.43(22.32-75.69) | 47.69(25.58-84.01) | 0.97(0.47-1.47) | |
| Germany | 85249.97(37272.89-131953.57) | 64.02(28.47-98.50) | 156415.89(73442.74-239926.23) | 68.48(32.21-105.01) | 83.48(56.54-118.61) | 0.38(0.29-0.47) | |
| Ghana | 1166.04(398.10-1911.70) | 27.63(9.43-45.81) | 3687.01(1519.74-5892.63) | 32.19(13.20-51.36) | 216.20(154.12-314.94) | 0.29(0.13-0.45) | |
| Greece | 5864.45(2328.77-9233.21) | 39.31(15.88-62.08) | 10048.00(4454.91-15883.60) | 34.53(15.59-53.93) | 71.34(45.37-112.38) | -0.75(-0.97--0.53) | |
| Greenland | 19.22(8.65-29.47) | 79.04(36.81-123.33) | 33.16(15.87-53.36) | 59.10(28.34-94.12) | 72.50(43.05-112.48) | -0.80(-0.92--0.67) | |
| Grenada | 29.09(10.84-46.92) | 35.94(13.53-58.03) | 45.60(21.01-71.44) | 47.14(21.25-74.38) | 56.76(24.86-113.93) | 0.87(0.65-1.08) | |
| Guam | 19.84(8.94-31.83) | 36.61(16.36-60.56) | 71.43(34.07-112.09) | 33.26(15.84-52.42) | 260.10(198.14-350.81) | 0.12(-0.07-0.31) | |
| Guatemala | 714.30(307.72-1168.88) | 28.74(11.98-46.57) | 3415.02(1603.73-5363.95) | 34.19(16.04-54.22) | 378.10(302.04-502.71) | 0.67(0.54-0.80) | |
| Guinea | 533.19(187.37-968.81) | 20.05(6.81-35.91) | 1126.25(423.44-1846.64) | 25.80(9.71-42.17) | 111.23(64.48-178.83) | 0.87(0.84-0.91) | |
| Guinea-Bissau | 68.89(25.36-115.83) | 25.69(9.03-43.95) | 144.05(56.16-238.52) | 32.70(12.55-54.38) | 109.09(65.27-169.53) | 0.83(0.79-0.88) | |
| Guyana | 109.86(43.24-179.45) | 34.75(13.64-56.77) | 227.55(101.49-369.06) | 42.20(18.62-68.21) | 107.12(71.89-166.83) | 0.75(0.59-0.90) | |
| Haiti | 876.38(299.36-1541.62) | 36.46(12.14-65.02) | 2270.82(805.83-3888.66) | 40.71(14.09-69.32) | 159.11(100.88-230.72) | 0.54(0.49-0.59) | |
| Honduras | 570.39(230.76-921.13) | 33.75(13.39-55.03) | 2681.19(1225.54-4310.24) | 52.26(23.75-84.54) | 370.06(257.62-532.38) | 1.49(1.31-1.66) | |
| Hungary | 7188.13(3309.90-10750.53) | 50.66(23.41-74.63) | 9016.13(4477.04-13390.22) | 42.47(21.18-63.36) | 25.43(13.20-43.95) | -0.49(-0.56--0.43) | |
| Iceland | 133.00(57.98-212.25) | 43.74(19.06-69.70) | 339.81(155.27-524.87) | 52.04(23.61-79.99) | 155.50(116.78-205.97) | 0.71(0.59-0.82) | |
| India | 66116.29(22835.15-116711.76) | 19.45(6.73-34.69) | 259335.48(94619.62-436668.53) | 26.16(9.64-43.91) | 292.24(236.93-365.56) | 1.08(0.99-1.18) | |
| Indonesia | 26537.12(9123.01-43263.75) | 37.21(12.57-60.53) | 86420.12(32366.71-139388.29) | 52.29(19.75-83.85) | 225.66(178.87-282.87) | 1.14(1.07-1.21) | |
| Iran (Islamic Republic of) | 3729.47(1529.13-6043.06) | 21.34(8.58-34.59) | 17194.14(8672.52-26235.68) | 25.99(13.02-39.70) | 361.03(274.86-525.24) | 0.54(0.45-0.62) | |
| Iraq | 2124.06(1000.92-3251.46) | 29.27(13.79-44.88) | 6947.19(3295.21-10981.96) | 39.46(18.92-62.19) | 227.07(154.30-344.94) | 0.71(0.60-0.82) | |
| Ireland | 2168.43(895.05-3346.60) | 53.28(21.96-82.55) | 3563.88(1563.27-5569.72) | 42.21(18.55-65.92) | 64.35(43.04-92.86) | -0.98(-1.18--0.78) | |
| Israel | 2555.32(1104.35-3972.77) | 53.90(23.13-83.50) | 7506.86(3414.13-11632.09) | 56.45(25.61-87.79) | 193.77(151.81-253.59) | 0.49(0.27-0.71) | |
| Italy | 42868.21(17026.63-68281.44) | 47.48(18.79-75.85) | 65857.21(30143.08-103826.99) | 37.10(16.53-58.57) | 53.63(31.07-85.57) | -1.07(-1.19--0.96) | |
| Jamaica | 597.24(237.12-979.52) | 32.29(12.90-52.88) | 1416.40(645.57-2214.54) | 43.03(19.77-67.62) | 137.16(97.93-214.34) | 1.04(0.94-1.13) | |
| Japan | 46785.46(16811.74-75295.51) | 28.69(10.29-45.99) | 83513.32(30750.66-140008.38) | 19.26(7.21-31.87) | 78.50(55.84-103.63) | -1.82(-2.19--1.44) | |
| Jordan | 235.17(115.39-360.19) | 24.46(11.65-37.30) | 1402.48(718.17-2127.07) | 25.91(13.27-39.02) | 496.36(376.38-656.02) | 0.05(-0.13-0.22) | |
| Kazakhstan | 3353.93(1457.58-5353.93) | 30.17(13.41-47.71) | 5646.33(2640.97-8669.30) | 37.54(17.85-56.46) | 68.35(44.36-100.22) | 0.50(0.38-0.61) | |
| Kenya | 1293.10(460.29-2247.09) | 19.56(6.88-33.77) | 4607.81(1787.91-7638.28) | 26.88(10.14-44.45) | 256.34(194.60-349.90) | 1.12(1.06-1.18) | |
| Kiribati | 6.96(3.07-11.46) | 22.71(9.70-38.30) | 18.40(9.59-29.32) | 32.98(16.11-52.70) | 164.25(103.80-253.42) | 1.11(1.01-1.20) | |
| Kuwait | 97.19(43.83-152.39) | 23.02(10.18-36.00) | 648.43(339.49-980.44) | 28.79(15.11-42.68) | 567.14(463.10-773.53) | 0.89(0.53-1.26) | |
| Kyrgyzstan | 591.57(253.43-951.82) | 21.96(9.37-34.96) | 1040.49(479.51-1583.90) | 26.15(11.82-39.63) | 75.89(47.07-120.95) | 0.60(0.51-0.69) | |
| Lao People's Democratic Republic | 436.12(153.08-747.72) | 29.43(10.43-51.28) | 1177.36(447.77-1986.63) | 34.49(13.14-59.22) | 169.96(112.64-254.03) | 0.53(0.51-0.56) | |
| Latvia | 1245.65(569.18-1917.93) | 35.30(16.34-54.20) | 2007.17(959.45-3029.62) | 45.29(21.75-68.56) | 61.13(37.15-90.15) | 1.06(0.93-1.19) | |
| Lebanon | 515.70(206.76-862.96) | 29.35(11.49-49.60) | 1672.92(803.00-2534.37) | 25.57(12.24-38.67) | 224.40(120.45-418.62) | -0.60(-0.75--0.45) | |
| Lesotho | 147.22(60.66-249.80) | 20.86(8.45-35.87) | 255.08(123.66-401.73) | 30.82(14.96-47.90) | 73.26(27.27-131.70) | 1.78(1.52-2.04) | |
| Liberia | 228.21(87.81-374.54) | 26.30(10.01-43.83) | 473.98(211.50-762.44) | 32.22(13.72-51.93) | 107.70(65.30-175.66) | 0.71(0.66-0.75) | |
| Libya | 352.87(151.31-566.06) | 21.84(9.11-34.92) | 1284.27(617.96-2079.21) | 30.23(14.41-49.22) | 263.95(176.36-427.29) | 1.32(1.20-1.45) | |
| Lithuania | 1628.69(733.69-2544.86) | 36.47(16.39-56.69) | 2962.02(1488.44-4404.71) | 46.11(22.72-69.57) | 81.87(58.46-117.09) | 0.81(0.73-0.90) | |
| Luxembourg | 293.45(125.12-447.83) | 53.91(22.95-82.27) | 597.75(283.85-915.55) | 50.97(24.14-78.16) | 103.70(74.95-143.03) | 0.03(-0.05-0.12) | |
| Madagascar | 965.69(297.77-1797.20) | 24.10(7.19-45.02) | 2547.19(916.08-4416.65) | 33.12(11.52-58.05) | 163.77(104.68-263.05) | 1.10(1.05-1.15) | |
| Malawi | 567.90(184.00-995.74) | 18.46(5.88-32.68) | 1513.44(529.98-2551.97) | 26.50(9.45-44.07) | 166.50(120.98-243.34) | 1.10(1.00-1.19) | |
| Malaysia | 2491.80(907.80-4054.58) | 30.93(11.12-50.93) | 10714.82(4490.43-16804.47) | 45.18(18.77-71.15) | 330.00(280.79-429.41) | 1.61(1.42-1.80) | |
| Maldives | 17.98(6.40-30.67) | 28.96(9.85-49.82) | 88.11(34.33-144.29) | 31.37(11.74-50.89) | 390.09(276.88-533.87) | 0.10(0.01-0.19) | |
| Mali | 503.10(176.61-878.44) | 19.37(6.86-33.42) | 1325.46(481.52-2220.80) | 22.37(8.18-37.14) | 163.46(110.61-233.68) | 0.54(0.47-0.61) | |
| Malta | 178.42(70.38-284.32) | 44.13(17.33-69.76) | 424.78(186.42-667.01) | 38.54(16.94-59.96) | 138.07(95.30-195.93) | -0.23(-0.37--0.08) | |
| Marshall Islands | 4.93(2.36-7.92) | 37.15(16.94-60.55) | 13.86(7.02-21.97) | 53.36(25.39-82.56) | 181.39(121.73-279.26) | 1.07(0.97-1.17) | |
| Mauritania | 232.56(99.84-367.79) | 30.57(13.34-48.26) | 634.17(299.74-984.36) | 38.41(17.99-59.72) | 172.69(111.71-257.24) | 0.61(0.54-0.68) | |
| Mauritius | 203.33(76.63-326.68) | 35.96(13.58-58.00) | 679.35(285.19-1059.74) | 39.98(16.97-62.43) | 234.11(198.26-310.98) | 0.11(-0.13-0.35) | |
| Mexico | 12428.68(5385.44-19780.20) | 36.01(15.75-57.84) | 51042.96(23621.74-77472.02) | 43.67(20.24-66.09) | 310.69(258.66-403.52) | 0.79(0.72-0.86) | |
| Micronesia (Federated States of) | 17.73(8.32-28.30) | 43.30(19.35-71.98) | 31.54(16.76-48.53) | 54.48(28.05-86.07) | 77.89(35.47-139.06) | 0.72(0.68-0.75) | |
| Monaco | 35.42(14.90-56.51) | 44.34(18.77-70.95) | 46.14(20.94-71.33) | 40.41(18.31-62.73) | 30.29(3.97-69.80) | -0.34(-0.42--0.26) | |
| Mongolia | 224.53(89.86-356.06) | 24.33(9.71-38.57) | 490.00(199.55-775.73) | 26.19(10.55-41.78) | 118.23(85.01-155.98) | 0.07(-0.02-0.16) | |
| Montenegro | 504.92(240.93-773.45) | 87.15(41.30-134.21) | 999.17(512.62-1529.34) | 112.49(56.04-173.21) | 97.89(57.88-160.65) | 1.03(0.80-1.27) | |
| Morocco | 2977.43(1146.73-4913.70) | 25.04(9.64-41.48) | 9189.54(4239.16-14433.22) | 32.60(14.78-50.84) | 208.64(150.14-298.61) | 0.84(0.78-0.91) | |
| Mozambique | 975.29(304.16-1771.97) | 20.41(6.31-37.30) | 2660.06(938.92-4636.19) | 31.58(11.15-54.96) | 172.75(117.93-252.92) | 1.62(1.52-1.73) | |
| Myanmar | 5402.29(1837.90-9241.43) | 31.73(10.56-55.02) | 14075.64(5071.90-23249.32) | 35.88(12.88-58.83) | 160.55(108.62-233.40) | 0.29(0.23-0.35) | |
| Namibia | 116.52(45.66-193.28) | 24.67(9.59-41.11) | 328.88(149.38-517.76) | 31.60(14.41-49.43) | 182.25(123.89-291.51) | 0.74(0.60-0.87) | |
| Nauru | 2.30(1.12-3.46) | 61.49(28.87-93.35) | 3.81(2.07-5.74) | 81.28(40.91-134.74) | 65.45(31.60-124.37) | 0.88(0.77-0.98) | |
| Nepal | 1084.60(315.89-2026.27) | 15.70(4.66-29.32) | 3466.31(1134.98-6310.58) | 18.27(5.74-34.04) | 219.59(147.96-320.58) | 0.56(0.40-0.71) | |
| Netherlands | 10317.68(4224.06-16432.32) | 49.95(20.35-79.44) | 18180.77(8030.94-28509.46) | 46.62(20.41-73.36) | 76.21(53.78-105.46) | -0.83(-1.12--0.55) | |
| New Zealand | 2379.73(1013.19-3618.16) | 62.08(26.36-94.85) | 4899.10(2263.99-7663.51) | 53.86(25.10-83.98) | 105.87(81.29-148.97) | -0.28(-0.45--0.11) | |
| Nicaragua | 429.24(195.20-693.35) | 32.51(14.68-52.45) | 1642.60(801.60-2578.31) | 37.62(18.40-58.80) | 282.68(225.61-369.51) | 0.62(0.50-0.73) | |
| Niger | 334.01(110.93-586.86) | 18.40(6.37-33.27) | 1217.83(393.76-2165.91) | 21.72(7.03-38.87) | 264.61(195.55-352.58) | 0.59(0.55-0.62) | |
| Nigeria | 7881.51(2974.41-13309.76) | 24.52(9.39-41.71) | 21498.06(8789.24-34247.95) | 33.31(13.46-52.74) | 172.77(111.58-259.35) | 1.14(1.05-1.23) | |
| Niue | 1.13(0.49-1.84) | 47.70(21.39-77.58) | 1.27(0.60-1.92) | 61.98(29.32-93.64) | 12.13(-11.72-44.89) | 0.68(0.58-0.78) | |
| North Macedonia | 709.85(323.04-1073.95) | 43.71(19.76-65.46) | 1505.09(699.41-2366.18) | 55.10(25.93-86.41) | 112.03(69.11-174.34) | 0.68(0.38-0.97) | |
| Northern Mariana Islands | 5.34(2.77-8.56) | 40.47(19.33-65.58) | 25.84(13.73-38.53) | 62.07(32.21-92.39) | 383.69(279.55-522.65) | 1.32(1.15-1.49) | |
| Norway | 4193.01(1718.08-6591.47) | 55.86(23.09-87.66) | 5156.59(2232.01-8164.02) | 45.37(19.62-71.76) | 22.98(10.53-36.46) | -0.87(-1.01--0.74) | |
| Oman | 100.65(39.45-171.38) | 19.17(7.34-32.56) | 511.51(265.45-783.65) | 36.65(19.37-55.84) | 408.20(239.37-688.17) | 2.80(2.52-3.08) | |
| Pakistan | 10840.78(3762.72-18744.41) | 23.26(8.13-40.33) | 30312.71(12119.94-49434.85) | 33.21(12.76-55.12) | 179.62(131.80-252.53) | 1.12(0.99-1.24) | |
| Palau | 2.59(1.22-4.14) | 31.64(15.10-51.15) | 7.71(3.74-11.71) | 43.85(21.53-65.96) | 198.33(139.05-272.81) | 1.06(0.95-1.17) | |
| Palestine | 188.81(85.82-294.70) | 27.77(12.34-43.84) | 559.11(283.72-831.65) | 32.38(16.57-48.38) | 196.13(128.96-301.78) | 0.33(0.20-0.45) | |
| Panama | 509.07(229.26-792.06) | 37.71(16.97-58.17) | 2052.28(1017.44-3164.73) | 45.27(22.31-70.07) | 303.14(245.84-391.67) | 0.65(0.61-0.69) | |
| Papua New Guinea | 228.56(71.95-426.78) | 17.12(5.05-31.84) | 909.67(343.77-1614.99) | 21.19(7.30-37.95) | 298.00(213.92-452.44) | 0.70(0.63-0.78) | |
| Paraguay | 784.62(315.50-1264.36) | 38.97(15.63-62.82) | 2563.08(1153.39-4079.38) | 47.63(21.24-75.72) | 226.67(167.78-334.10) | 0.79(0.72-0.87) | |
| Peru | 2130.02(840.70-3740.75) | 19.74(7.71-34.61) | 10710.65(4521.06-17339.77) | 32.39(13.67-52.29) | 402.84(292.74-587.08) | 1.94(1.73-2.15) | |
| Philippines | 6419.41(2392.11-11015.28) | 30.61(11.27-52.30) | 20505.87(8144.23-34505.31) | 31.48(12.03-53.72) | 219.44(169.98-289.95) | 0.13(0.07-0.19) | |
| Poland | 22454.60(10362.08-33973.12) | 54.25(25.22-82.66) | 35670.16(16843.67-55135.14) | 46.56(21.91-71.76) | 58.85(35.19-86.10) | -0.55(-1.03--0.07) | |
| Portugal | 5225.90(1977.55-8465.20) | 39.62(14.97-63.62) | 9359.15(4122.81-14789.38) | 32.37(13.98-51.48) | 79.09(50.36-127.44) | -0.71(-0.82--0.61) | |
| Puerto Rico | 1429.98(664.59-2251.72) | 42.42(19.58-66.94) | 3316.14(1557.87-5090.06) | 39.96(19.24-61.21) | 131.90(97.55-183.36) | -0.24(-0.29--0.18) | |
| Qatar | 22.02(9.51-35.46) | 41.77(15.40-69.13) | 174.11(89.71-285.55) | 34.14(15.64-55.33) | 690.60(467.32-1095.58) | -1.02(-1.49--0.55) | |
| Republic of Korea | 6484.58(2110.95-11572.46) | 27.19(8.91-47.78) | 21662.07(7753.70-38408.17) | 23.08(8.27-40.97) | 234.06(129.22-349.84) | -0.87(-1.02--0.72) | |
| Republic of Moldova | 1361.05(609.52-2126.21) | 36.40(16.66-56.26) | 2715.09(1341.06-4003.89) | 44.67(21.98-66.04) | 99.49(76.36-136.11) | 0.65(0.54-0.76) | |
| Romania | 9536.08(4077.30-14646.72) | 38.37(16.46-58.59) | 13446.08(6697.88-19712.02) | 33.04(16.56-48.86) | 41.00(17.53-71.88) | -0.82(-0.98--0.67) | |
| Russian Federation | 61421.21(26886.99-94645.20) | 37.62(16.32-57.69) | 111165.57(53828.41-169034.96) | 45.43(21.94-68.84) | 80.99(59.72-112.38) | 0.60(0.49-0.71) | |
| Rwanda | 454.49(134.74-837.46) | 21.28(6.20-39.70) | 1024.17(309.42-1890.36) | 21.95(6.50-40.70) | 125.34(67.51-211.69) | -0.32(-0.50--0.15) | |
| Saint Kitts and Nevis | 17.57(7.10-28.41) | 49.78(20.07-80.51) | 27.85(12.96-44.04) | 51.46(24.06-81.42) | 58.52(28.55-106.25) | 0.30(0.18-0.42) | |
| Saint Lucia | 41.75(15.16-66.69) | 57.73(20.39-91.82) | 106.05(44.66-173.50) | 45.82(19.13-75.29) | 154.01(110.92-224.49) | -1.01(-1.24--0.77) | |
| Saint Vincent and the Grenadines | 26.88(9.96-44.88) | 41.19(14.91-68.96) | 56.71(23.77-92.10) | 43.51(18.60-69.88) | 111.02(77.60-167.12) | 0.29(0.13-0.45) | |
| Samoa | 31.27(14.43-48.94) | 42.89(19.27-67.79) | 68.91(37.43-105.62) | 54.41(28.27-84.25) | 120.32(83.78-174.10) | 0.72(0.68-0.76) | |
| San Marino | 18.67(7.98-29.15) | 50.06(21.30-78.73) | 30.92(13.10-48.61) | 34.28(14.84-53.47) | 65.65(30.47-118.44) | -0.91(-1.09--0.73) | |
| Sao Tome and Principe | 12.83(4.78-21.22) | 23.46(8.49-39.35) | 28.89(12.40-45.28) | 35.32(14.98-55.60) | 125.22(77.95-197.50) | 1.49(1.44-1.54) | |
| Saudi Arabia | 948.87(405.75-1504.70) | 21.35(9.24-33.86) | 3625.78(1942.69-5495.38) | 29.65(15.62-44.27) | 282.12(188.52-455.11) | 0.98(0.91-1.05) | |
| Senegal | 685.73(262.34-1134.91) | 28.01(10.68-45.90) | 2013.35(795.92-3237.87) | 34.89(13.61-55.49) | 193.61(136.09-274.45) | 0.62(0.58-0.66) | |
| Serbia | 4315.91(1836.08-6624.98) | 48.88(20.66-75.52) | 6938.93(3425.45-10299.74) | 39.66(19.70-58.53) | 60.78(33.28-106.54) | -0.97(-1.21--0.72) | |
| Seychelles | 21.09(7.96-33.65) | 37.99(14.32-60.81) | 42.94(19.81-69.15) | 42.97(19.25-69.89) | 103.67(68.64-163.80) | 0.52(0.42-0.62) | |
| Sierra Leone | 495.17(174.78-825.25) | 29.84(10.61-49.28) | 882.99(337.13-1404.55) | 31.16(12.06-49.51) | 78.32(50.32-121.64) | 0.08(0.05-0.10) | |
| Singapore | 413.30(134.36-703.00) | 21.11(6.98-36.16) | 1045.35(422.83-1789.11) | 12.35(4.97-21.06) | 152.92(102.61-236.40) | -2.29(-2.58--2.01) | |
| Slovakia | 3634.36(1741.14-5379.91) | 62.44(29.97-91.72) | 6179.16(3081.22-9246.17) | 63.37(31.68-94.99) | 70.02(41.39-102.76) | 0.10(-0.01-0.20) | |
| Slovenia | 999.02(459.85-1526.27) | 40.83(18.96-62.32) | 1935.00(921.20-2926.83) | 38.63(18.59-58.54) | 93.69(63.04-126.98) | -0.03(-0.26-0.19) | |
| Solomon Islands | 21.52(8.04-39.36) | 21.13(7.59-38.61) | 80.79(35.29-137.63) | 30.95(11.99-54.75) | 275.42(195.56-406.90) | 1.17(1.09-1.25) | |
| Somalia | 265.01(78.86-495.41) | 15.93(4.66-30.45) | 799.79(263.10-1491.65) | 18.88(6.15-35.27) | 201.80(131.21-309.20) | 0.68(0.60-0.77) | |
| South Africa | 5176.68(2214.69-8181.42) | 28.96(12.33-45.42) | 14717.75(7300.10-22051.68) | 38.44(18.90-57.26) | 184.31(146.10-243.97) | 0.90(0.69-1.10) | |
| South Sudan | 372.39(103.60-703.67) | 17.70(4.99-33.33) | 626.80(202.41-1151.87) | 21.83(7.00-40.26) | 68.32(29.38-123.46) | 0.71(0.67-0.74) | |
| Spain | 27842.13(11927.55-43392.35) | 50.69(21.74-79.10) | 50824.98(23854.18-80013.28) | 43.67(20.81-68.58) | 82.55(55.99-124.25) | -0.43(-0.53--0.33) | |
| Sri Lanka | 2164.91(777.68-3643.71) | 26.84(9.48-44.41) | 7975.81(3087.94-13174.71) | 33.33(12.93-54.67) | 268.41(194.03-380.32) | 1.12(0.93-1.30) | |
| Sudan | 1659.82(666.41-2835.68) | 22.83(9.28-39.53) | 4569.36(2217.75-7175.75) | 30.10(14.32-47.07) | 175.29(112.46-291.27) | 0.93(0.85-1.00) | |
| Suriname | 77.10(27.78-131.09) | 34.09(11.98-58.05) | 198.54(85.21-322.92) | 33.66(14.34-54.45) | 157.52(99.89-258.45) | 0.11(0.01-0.20) | |
| Sweden | 8642.01(3513.12-13831.25) | 51.73(20.97-82.74) | 20455.87(9160.21-32536.08) | 78.50(35.06-125.54) | 136.70(103.66-183.23) | 1.31(1.15-1.47) | |
| Switzerland | 2610.35(1086.71-4241.55) | 23.14(9.65-37.64) | 4786.12(2164.76-7718.00) | 22.10(10.00-35.54) | 83.35(52.62-125.77) | 0.30(0.03-0.56) | |
| Syrian Arab Republic | 1098.26(487.55-1788.04) | 27.05(11.73-44.34) | 3371.58(1773.56-5126.97) | 34.86(17.75-54.12) | 206.99(128.50-360.97) | 0.57(0.46-0.69) | |
| Taiwan (Province of China) | 3992.85(1410.08-6648.34) | 34.01(11.96-56.03) | 12093.95(5197.99-19711.70) | 27.23(11.73-44.48) | 202.89(158.17-284.78) | -0.94(-1.21--0.67) | |
| Tajikistan | 457.66(194.60-747.79) | 18.53(7.86-30.02) | 944.21(401.33-1540.14) | 20.39(8.78-32.77) | 106.31(74.55-147.27) | 0.31(0.19-0.44) | |
| Thailand | 6963.72(2367.95-12438.54) | 26.02(9.01-46.21) | 31792.46(12462.70-53511.24) | 29.07(11.39-48.82) | 356.54(251.13-534.47) | 0.16(-0.01-0.32) | |
| Timor-Leste | 50.14(17.06-85.74) | 25.96(8.66-43.56) | 223.25(79.28-372.36) | 32.51(11.66-54.40) | 345.20(253.53-459.93) | 0.88(0.78-0.98) | |
| Togo | 233.53(86.69-384.50) | 27.46(10.22-45.41) | 826.90(326.24-1343.09) | 34.25(13.59-53.72) | 254.08(192.37-363.34) | 0.69(0.63-0.75) | |
| Tokelau | 0.48(0.20-0.80) | 38.08(16.15-65.23) | 0.82(0.38-1.32) | 55.56(25.42-88.85) | 72.62(31.24-145.24) | 1.17(1.03-1.31) | |
| Tonga | 16.10(7.44-25.92) | 32.49(14.63-52.97) | 38.03(20.08-58.70) | 49.46(26.21-76.79) | 136.22(91.46-196.21) | 1.37(1.24-1.51) | |
| Trinidad and Tobago | 334.34(138.22-525.47) | 47.55(19.73-74.02) | 899.33(431.96-1410.39) | 47.97(23.09-75.47) | 168.99(124.36-240.03) | -0.01(-0.08-0.06) | |
| Tunisia | 771.43(298.00-1267.39) | 20.56(7.86-33.83) | 3126.93(1507.67-5145.21) | 27.30(13.20-45.55) | 305.34(206.87-497.43) | 0.80(0.76-0.84) | |
| Turkmenistan | 393.02(167.95-625.96) | 24.09(10.32-38.91) | 1145.11(509.95-1821.07) | 33.57(14.74-52.86) | 191.36(144.11-262.46) | 1.04(0.96-1.12) | |
| Tuvalu | 1.96(0.90-3.21) | 35.72(16.10-59.19) | 4.85(2.43-7.38) | 54.62(27.54-84.27) | 148.13(99.43-223.77) | 1.29(1.15-1.43) | |
| Türkiye | 6919.08(3176.22-10766.16) | 25.01(11.42-39.17) | 21296.30(11181.11-32482.26) | 25.69(13.41-38.96) | 207.79(135.85-307.20) | 0.00(-0.24-0.25) | |
| Uganda | 1189.38(361.84-2134.02) | 23.23(7.09-41.70) | 2690.38(896.35-4715.57) | 23.57(7.56-41.45) | 126.20(80.98-183.78) | -0.21(-0.32--0.10) | |
| Ukraine | 24713.13(11232.90-38272.02) | 36.80(16.62-56.17) | 33017.11(15711.06-49950.96) | 41.19(19.57-61.71) | 33.60(10.71-63.47) | 0.22(0.13-0.31) | |
| United Arab Emirates | 86.98(37.03-137.16) | 30.19(12.57-48.56) | 718.72(364.91-1141.80) | 35.04(17.89-52.01) | 726.26(490.66-1179.29) | 2.22(1.62-2.82) | |
| United Kingdom | 46514.93(20177.47-70709.95) | 48.81(21.30-73.92) | 59881.99(29414.22-91989.22) | 40.66(19.85-62.35) | 28.74(14.79-50.41) | -0.87(-1.10--0.64) | |
| United Republic of Tanzania | 1796.26(572.74-3133.71) | 21.60(6.84-38.42) | 5506.59(2138.89-9323.75) | 26.40(10.08-45.15) | 206.56(145.50-314.75) | 0.60(0.39-0.80) | |
| United States of America | 146973.01(65708.93-228968.84) | 43.91(19.65-68.10) | 330165.66(172922.64-504495.14) | 52.62(27.53-80.35) | 124.64(85.73-190.66) | 0.37(0.26-0.48) | |
| United States Virgin Islands | 32.14(14.07-50.44) | 50.07(22.26-78.21) | 73.87(34.01-115.62) | 41.22(19.24-64.76) | 129.81(89.53-181.94) | -0.48(-0.60--0.36) | |
| Uruguay | 1037.81(444.49-1664.99) | 26.92(11.64-42.95) | 1942.65(931.73-3018.81) | 30.14(14.47-47.09) | 87.19(57.27-128.25) | 0.39(0.32-0.45) | |
| Uzbekistan | 1625.01(630.23-2829.92) | 15.31(5.94-26.70) | 4755.61(2079.32-7751.93) | 21.31(9.17-34.51) | 192.65(133.06-269.90) | 1.19(1.11-1.26) | |
| Vanuatu | 19.77(7.41-32.94) | 41.91(15.91-69.15) | 67.12(29.10-106.20) | 48.66(20.14-76.77) | 239.56(180.91-323.99) | 0.38(0.32-0.44) | |
| Venezuela (Bolivarian Republic of) | 3712.03(1560.34-5770.37) | 44.63(18.48-69.27) | 12725.72(5947.12-20061.45) | 45.70(21.41-72.12) | 242.82(191.51-330.91) | -0.06(-0.17-0.04) | |
| Viet Nam | 9498.68(3074.70-16189.77) | 27.98(9.14-47.85) | 33412.19(11767.73-54621.08) | 41.43(14.67-67.96) | 251.76(185.56-334.98) | 1.53(1.40-1.65) | |
| Yemen | 634.26(227.86-1154.02) | 18.80(6.70-33.43) | 2488.25(1100.13-4017.21) | 24.37(10.70-39.99) | 292.31(186.27-481.54) | 0.81(0.73-0.89) | |
| Zambia | 406.60(130.82-735.27) | 19.45(6.11-34.74) | 1691.22(626.11-3175.95) | 32.07(11.61-59.13) | 315.94(164.34-579.82) | 1.80(1.52-2.07) | |
| Zimbabwe | 711.95(256.99-1157.37) | 24.02(8.54-39.35) | 1640.33(724.51-2584.76) | 32.71(13.48-50.73) | 130.40(79.56-207.87) | 1.27(0.98-1.56) | |

^AF/AFL, atrial fibrillation/atrial flutter; EAPC, estimated annual percentage changes; SDI, Socio-Demographic Index; GBD, Global Burden of Disease, Injuries, and Risk Factors Study; ASR, age standardized rate; UI, uncertainty interval; PC, percentage change.^
